# A cellular expression map of epidermal and subepidermal cell layer‐enriched transcription factor genes integrated with the regulatory network in Arabidopsis shoot apical meristem

**DOI:** 10.1002/pld3.306

**Published:** 2021-03-18

**Authors:** Shivani Bhatia, Harish Kumar, Monika Mahajan, Sonal Yadav, Prince Saini, Shalini Yadav, Sangram Keshari Sahu, Jayesh Kumar Sundaram, Ram Kishor Yadav

**Affiliations:** ^1^ Department of Biological Sciences Indian Institute of Science Education and Research Mohali Punjab India; ^2^ Present address: Department of Biological Sciences University of Pittsburgh Pittsburgh PA USA

**Keywords:** Cell layer, epidermal, gene regulatory network, shoot apical meristem, subepidermal, transcription factors

## Abstract

Transcriptional control of gene expression is an exquisitely regulated process in both animals and plants. Transcription factors (TFs) and the regulatory networks that drive the expression of TF genes in epidermal and subepidermal cell layers in Arabidopsis are unexplored. Here, we identified 65 TF genes enriched in the epidermal and subepidermal cell layers of the shoot apical meristem (SAM). To determine the cell type specificity in different stages of Arabidopsis development, we made YFP based transcriptional fusion constructs by taking a 3‐kb upstream noncoding region above the translation start site. Here, we report that for ~52% (22/42) TF genes, we detected transcription activity. TF genes derived from epidermis show uniform expression in early embryo development; however, in the late globular stage, their transcription activity is suppressed in the inner cell layers. Expression patterns linked to subepidermal cell layer identity were apparent in the postembryonic development. Potential upstream regulators that could modulate the activity of epidermal and subepidermal cell layer‐enriched TF genes were identified using enhanced yeast‐one‐hybrid (eY1H) assay and validated. This study describes the activation of TF genes in epidermal and subepidermal cell layers in embryonic and postembryonic development of Arabidopsis shoot apex.

## 1 INTRODUCTION

One of the challenges in developmental biology is to understand how the expression pattern of TFs linked to cell fate specification and tissue specialization are established and maintained. In angiosperms, cell fate specification events first take place in embryonic development. Lateral organs are formed in the shoot apex in postembryonic development. Cell type specifications occur independently in the newly formed organs with barring a few exceptions (Barton, 2010). Majority of plant organs in postembryonic development maintain the identity of epidermal, subepidermal, and vascular cell types. Besides, postembryonic SAM is also divided into zones based on the cell identities and cell behavior (Steeves & Sussex, 1989). The cells at the center of the meristem summit are identified as stem cells/central zone (CZ) because of the infrequent nature of cell division in them. When CZ cells divide, they enter into the surrounding peripheral zone (PZ), where cell division occurs at a regular interval. Eventually, cells leave the meristem and become part of the new organs (Meyerowitz, 1997). Stem cells pushed beneath the CZ form rib meristem (RM), which give rise to stem and vascular cell types. Studies in the past identified genes whose expression patterns were tightly linked to stem cells and epidermal cell type in Arabidopsis SAM (Abe et al., 2001; Aggarwal et al., 2010; Fletcher et al., 1999). However, expression patterns relating to the function of subepidermal and corpus cell types in SAM are not known.

After fertilization, in Arabidopsis, the first division of zygote produces a smaller apical cell and a large basal cell. The upper cell gives rise to a four‐cell embryo after two rounds of longitudinal cell division. A transverse division in the embryo leads to the formation of an octant with upper and lower tiers of cells. The resulting octant would undergo tangential cell division and give rise to an equal number of inner and outer cells. This division also separates the protoderm (future epidermis) from the inner cells. Later, a series of rapid cell divisions in the apical cell gives rise to the embryo proper in which epidermal, subepidermal, and vascular cell type identities are established to form a functional shoot and root apical meristem (Möller & Weijers, 2009). Thus, the stereotypical cell division pattern and cell fate specification events that took place in the early embryo are controlled precisely by regulatory networks.

TFs belonging to the HD‐ZIP IV family, *ARABIDOPSIS THALIANA MERISTEM LAYER 1* (*ATML1*) and *PROTODERMAL FACTOR 2* (*PDF2*) are preferentially retained in the epidermis and promote epidermal cell identity in embryonic and postembryonic development (Abe et al., 2001, 2003). In addition to *ATML1* and *PDF2*, high‐resolution transcriptome studies based on the fluorescent activated cell sorting, translatome, and nuclei sorting method have identified genes that are exclusively expressed in the epidermal cell layer in both embryonic and postembryonic development (Palovaara et al., 2017; Tian et al., 2019; Yadav et al., 2014). Despite their high‐resolution nature, often gene expression studies miss vital information related to spatial and temporal dynamics of gene expression in both plants and animals. We still do not know, when and wherein the epidermis and subepidermis, genes related to their functions are activated or repressed in the development.

By constructing the gene regulatory network (GRN), we can elucidate how the activation and repression of genes occur in cell and tissue‐specific manner. GRNs can also explain the spatiotemporal dynamics of gene regulation, which is vital for understanding the mechanisms of development in higher organisms. It is essential to identify the upstream TFs that interact with the cognate genomic region present within the promoter to construct the GRN. Advances in TF centered approaches such as DAP‐seq and chromatin immunoprecipitation followed with sequencing (ChIP‐seq) has enabled us to map protein DNA interaction (PDI) at genome‐scale in Arabidopsis (Lai et al., 2019). However, these techniques are highly successful for broadly expressed TFs. Interestingly, gene‐centered approaches developed as a useful tool to map the PDIs in both animals and plants, where high‐quality antibodies are challenging to generate against the TF proteins. Several studies successfully mapped large‐scale interaction between the genomic regions and TFs to delineate the GRNs using gene‐centered approaches (Deplancke, Mukhopadhyay, et al., 2006; Taylor‐Teeples et al., 2015).

Here, we report a cellular expression map for epidermal and subepidermal cell layer‐enriched TF genes. We observed that a majority of the epidermal cell layer‐enriched genes once get restricted to the epidermis in embryonic development retain it in postembryonic development. Expression patterns linked to the subepidermal cell layer are only discernible in postembryonic development. To elucidate the TF regulatory network, we have used eY1H assay. By combining the network biology approaches with expression patterns, we deciphered the role of upstream factors in controlling the activity of epidermal and subepidermal cell layer‐enriched TF genes in Arabidopsis.

## 2 RESULTS

### 2.1 Identification of epidermal and subepidermal cell layers‐enriched genes encoding TFs

To dissect the GRN that underlies epidermal and sub‐epidermal cell layers specification and maintenance in SAM, we mined the high‐resolution gene expression data from epidermal, sub‐epidermal, and corpus cell layers of SAM. This dataset was generated from sorted cells, for which epidermal/L1 cell layer, subepidermal/L2 cell layer, and corpus/L3 cell layer were labeled by fluorescent protein‐based reporter driven by the promoter of *ARID‐HMG DNA‐BINDING PROTEIN 15* (*ATHMGB15*), *HOMEODOMAIN GLABROUS 4* (*HDG4*) and *WUSCHEL* gene, respectively (Yadav et al., 2014). We identified the transcripts that show a ≥1.5‐fold higher expression (*p* < 0.01) in the target cell layer compared with the other cell layers in the group. Of the 1,456 genes identified as differentially expressed across three cell layers, 535 were enriched in the epidermal/L1 layer, 256 in the subepidermal/L2 layer, and 665 in the L3/corpus. Of the 535 L1 layer‐enriched transcripts, 44 encode TFs, of the 256 L2 layer‐enriched transcripts, 21 encode TFs. We selected 44 TF encoding genes from the epidermis and 21 TF encoding genes from the subepidermis in this study to construct the GRNs (Figure 4a).

### 2.2 Generation of reporter lines

To create an atlas of spatiotemporal gene expression pattern for epidermal and subepidermal‐enriched TF genes, we made transcriptional fusion constructs. To achieve up to the limit of the native expression pattern, we choose 3 kb promoter DNA fragment from WT‐L*er*. The promoters were assembled in a gateway compatible binary vector containing the yellow fluorescent protein (YFP) translationally fused to HISTONE H2B (H2B) at the N‐terminus (H2B‐YFP). Of the 65 TFs selected, transgenics were obtained for 42. We chose H2B because the H2B‐YFP translational fusion protein ends up in the cell nucleus and gives a robust fluorescent signal that is easily detected in the given tissue using confocal microscopy at single‐cell resolution. For each promoter, multiple independent first‐generation (T1) transgenic lines were selected and screened under the upright fluorescence microscope after clipping off the old flower buds to see the expression of promoter‐reporter in the shoot apex and flower primordia. Further, the shoot apices of T1 plants were scanned under the confocal microscope to determine the spatiotemporal expression pattern.

From the T1 lines, two representative lines were selected and analyzed by confocal microscopy to establish a pattern of expression for various cell and tissue types after making them homozygote for the transgene (Table S1). For the ~50% (22/42) TF genes, a characteristic expression pattern was observed in the shoot apex and developing flowers. A detailed analysis of the transformants selected for each construct and primers used for amplifying the 3 kb promoter DNA fragment are given in Tables S1, S4 and S10. Promoter‐reporters of *CBF1* and *WRKY25* showed sporadic expression in the SAM (Figure S1). For 18 TF gene promoters, we did not see the expression in the selected transgenic plant lines, suggesting that the 3‐kb upstream noncoding sequence is insufficient to drive the expression of reporter gene. It is possible that the basal activity of the promoter did not result in a sufficient amount of H2B‐YFP protein. Thus, it was not visible to the eyes when the selected lines were examined under the fluorescence microscope.

### 2.3 TFs showing expression in the early embryo

**FIGURE 1:**
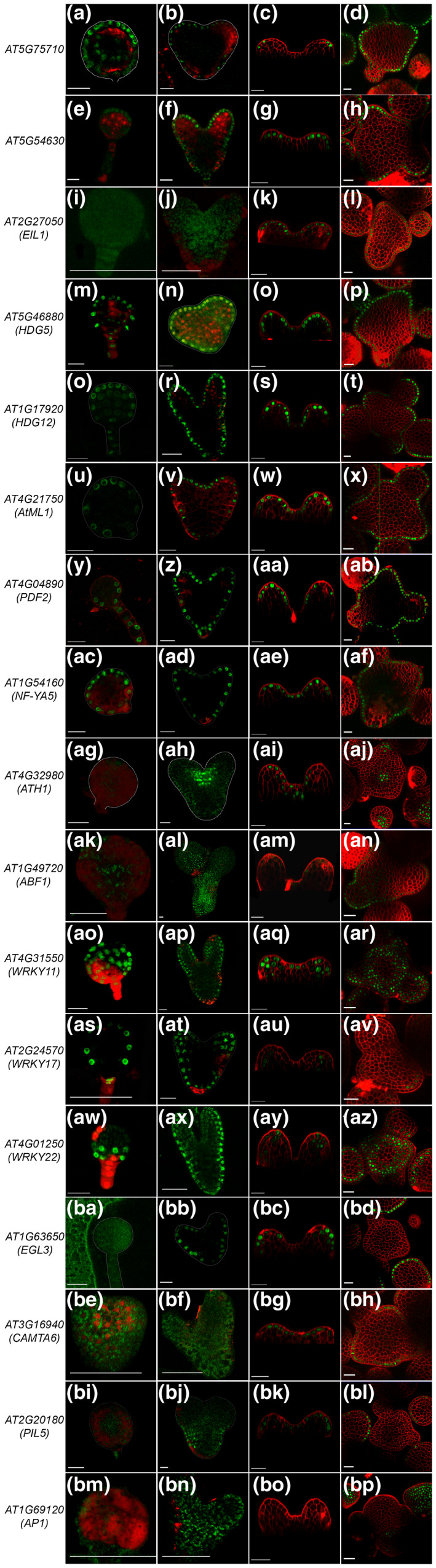
Spatiotemporal expression pattern revealed for epidermal cell layer enriched TF genes in embryonic and postembryonic development in Arabidopsis. From left to right the picture represents globular, heart stage, 3 DAG seedlings and inflorescence meristems (IMs), respectively. The Arabidopsis gene identifier and gene name are written for each promoter reporter construct on the left. Of the 17 TF promoter‐reporters studied in IM (d, h, l, p, t, x, ab, af, aj, an, ar, av, az, bd, bh, bl, bp ), ten are active in globular stage embryo (a, e, m, q, u, y, ac, ao, as and aw) followed with 14 in heart stage (b, f, n, r, v, z, ad, ah, al, ap, at, ax, bb and bj), 16 in 3 DAG seedlings (c, g, k, o, s, w, aa, ae, ai, am, aq, au, ay, bc, bg and bk. Scale bars = 20 μM

**FIGURE 2:**
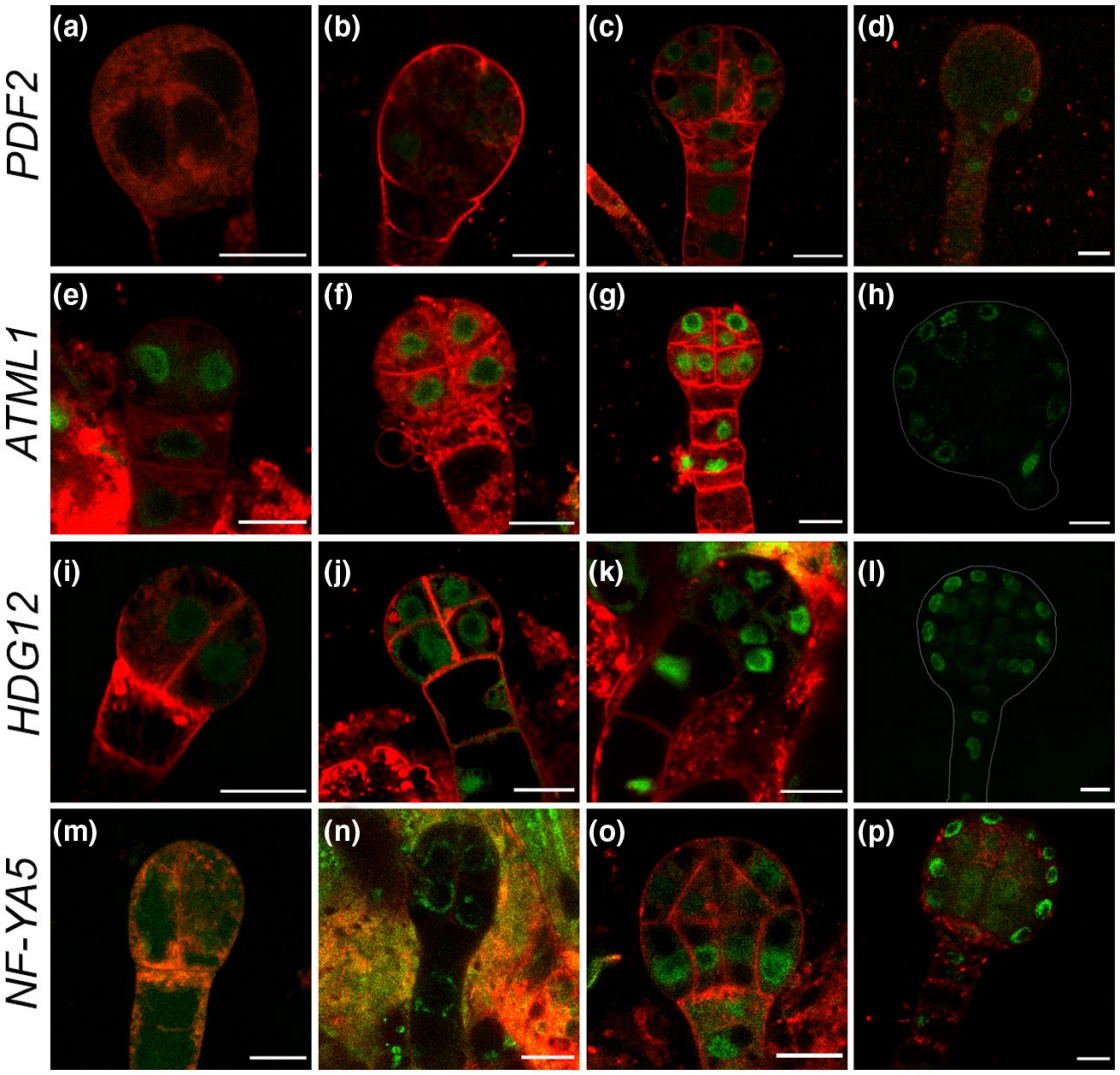
Repression of epidermal cell layer enriched TF genes occur in the inner layers in late globular stage. In the four‐cell and eight‐cell stage of embryos,*PDF2* expression is not detected (a and b). *PDF2* expresses uniformly in early globular stage embryo (c), in globular stage its expression is visible within the protoderm (d). In contrast, *ATML1* expression is seen from 4‐cell stage onward, and its expression get restricted to outer cell layer in the late globular stage (e–h). *HDG12* and *NF‐YA5* expression also show a similar expression pattern (i–l and m–p). *HDG12* expression at four‐cell (i), eight‐cell (j), 16‐cell (k) and early globular stage (l). (m‐p) *NF‐YA5* expression at four‐cell (m), eight‐cell (n), 16‐cell (o), and early globular stage (p). Scale bars = 10 μM

None of the past studies had investigated the expression pattern of epidermal and subepidermal cell layer‐enriched TF genes in a systematic and unbiased manner to understand their dynamic expression pattern in embryonic and postembryonic development. First, we studied the dynamic regulation of 22 TF genes in globular and heart stages of embryo development. Promoter::H2B‐YFP lines established based on the SAM expression pattern were used for this analysis. We found the expression of *AT1G75710, HOMEODOMAIN GLABROUS 5* (*HDG5*), *HDG12*, *ATML1*, *PDF2,* and *NUCLEAR FACTOR YA5* (*NF‐YA5*) restricted to the epidermal cell layer in globular stage embryo (Figure 1a, m, q, u, y, ac). An embryo goes through different stages of development; therefore, to precisely capture the signature of protoderm cell fate specification in early embryo we choose *PDF2*, *ATML1*, *HDG12,* and *NF‐YA5* lines. We examined the reporter activity starting from the 4‐cell stage onward for these genes (Figure 2a–p). We found a uniform expression of reporter protein (H2B‐YFP) in the 16‐cell stage embryo; however, in the 64‐cell stage embryo the expression of *PDF2*, *ATML1*, *HDG12,* and *NF‐YA5* was retained strongly in the outer cell layer (protoderm) (Figure 2d,h,l,p). A similar observation was also made using a 3.4 kb promoter fragment for *ATML1* by Takada et al., (2007). Taken together, the results obtained here not only supports the previous findings but also indicate that TF genes involved in protoderm cell fate specification and its function show specificity by the 64‐cell stage of embryo development. If one removes presumptive protoderm cells before the 64‐cell stage, protoderm may be respecified again from the inner cells.

One gets a notion looking at the high‐resolution cell population transcriptome data that the enrichment of a gene when predicted to a specific cell type, its reporter activity should entirely be confined to that cell type. However, advances in single‐cell genomics can differentiate cells that are not in sync with the rest of the cell populations. *AT5G54630* show enrichment in the epidermal cell layer. Despite that its expression is confined to the protoderm of the apical region of the embryo (Figure 1e‐h). The expression pattern of *AT5G54630* is restricted to the adaxial side of the cotyledon primordia in the heart stage (Figure 1f). *AT5G54630* could serve as a vital marker gene for apical fate studies. *ARABIDOPSIS THALIANA HOMEOBOX PROTEIN1* (*ATH1*), *ENHANCER OF GLABRA 3* (*EGL3*), and *PHYTOCHROME INTERACTING FACTOR 3‐LIKE 5* (*PIL5*) did not show expression in the globular stage embryo; however, in the heart stage their promoter activity became functional. *ATH1* expression was detected in the presumptive stem cell niche of heart stage embryo (Figure 1ah). *EGL3*, expression was observed again in the epidermal cell layer (Figure 1bb). In contrast, *PIL5* was active in the presumptive hypocotyl region of the embryo (Figure 1bj). Taken together with the results, we confirm that *ATH1* and *PIL5* are not expressed in the early embryo epidermis at all, and thus, deviate from their predicted expression pattern.

In the cell type‐specific transcriptome data, we found *WRKY11*, *WRKY17,* and *WRKY22* enriched in the epidermal cells (Yadav et al., 2014). However, *pWRKY11::H2B‐YFP* was active in the globular stage while in the heart stage, its expression got restricted to the epidermis and subepidermis (Figure 1ao, ap). In contrast, *WKRY17* was restricted to the epidermal cell layer in the globular stage. In the heart stage, its expression expanded toward the inner cell layers (Figure 1as, at). *WKRY22* was active in the lower tier of octant embryo. In the late heart stage, its expression got extended toward the cotyledons (Figure 1aw, ax). Taken together with the reporter activity of *WRKY11, WRKY17,* and *WRKY22*, we conclude that the activity of a particular gene in space and time may vary in a given tissue. WRKY family TF genes are highly unpredictable in their expression pattern in different stages of embryo development.

### 2.4 Epidermal cell layer‐enriched TF genes activated in postembryonic development

To understand the regulation of epidermal cell layer‐enriched TFs in postembryonic development, we examined the TF promoter‐reporters in 3‐days after germination (DAG) seedlings and 4‐week‐old plants, respectively. The expression pattern of two closely related C2H2 zinc finger genes; *AT1G75710* and *AT5G54630* resembled very closely in the seedling and adult SAM (Figure 1c, d, g, h). Although both the TF genes showed a departure from their embryonic expression patterns. In the heart stage, *pAT1G75710::H2B‐YFP* and *pAT5G54630::H2B‐YFP* showed expression restricted to the epidermis in the apical region. In the seedling, epidermal expression pattern disappeared from CZ but got restricted to the PZ cells in the adaxial epidermis.

HD‐ZIP IV family TF genes, e.g., *ATML1*, *PDF2*, *HDG5,* and *HDG12* are expressed in the epidermal cell layer in seedlings and inflorescence meristem (IM) (Figure 1o, p, s, t, w, x, aa, ab) (Abe et al., 2003; Nakamura et al., 2006). The *NF‐YA5* expression pattern was very similar to *ATML1* in seedling and adult SAM (Figure 1ae, af). *EGL3* and *PIL5* are expressed predominately in the epidermal cell layer of three DAG seedling; however, in adult plant SAM, both reporters showed expression mainly in flower primordium (Figure 1bd, bl). *ETHYLENE‐INSENSITIVE 3‐LIKE1* (*EIL1*) and *CALMODULIN BINDING TRANSCRIPTION ACTIVATOR6* (*CAMTA6*) are expressed in the epidermal cell layer post‐germination (Figure 1k, l, bg, bh). Recent studies indicated the role of *EIL1* and *ETHYLENE INSENSITIVE3* in apical hook development in seedlings (Zhang et al., 2018), while *CAMTA6* is implicated in Na^+^ homeostasis during germination (Shkolnik et al., 2019). Transcription activation of *pEIL1* and *pCAMTA6* is consistent with their function in seedling development.

*ABCISIC ACID RESPONSIVE ELEMENT‐BINDING FACTOR 1* (*ABF1*), *ATH1, WRKY11*, *WRKY17,* and *WRKY22* are enriched in the epidermal cell layer. *ABF1* reporter showed expression activity in epidermal, sub‐epidermal, and corpus cell layer in the shoot apex and young flower buttress. In stage‐2 flower, *pABF1* activity was restricted to the epidermal cell layer. *pATH1* expression pattern was overlapped partially in shoots and flowers (Gomez‐Mena & Sablowski, 2008). The 3 kb promoter used for *ATH1* transcriptional reporter did not recapitulate the native messenger RNA expression pattern (Figure 1aj) Interestingly, promoter‐reporter lines for *WRKY11, WRKY17,* and *WRKY22* showed broader expression in comparison to the predicted one (Yadav et al., 2014).

Two closely related MADS‐box TFs, *APETALA 1* (*AP1*) and *CAULIFLOWER* are found to be enriched in the epidermal cell layer in the cell type microarray study. Past studies have shown that these TFs are expressed in emerging flower primordia (Alejandra Mandel et al., 1992; Kempin et al., 1995; Ye et al., ). Confocal imaging of *pAP1::H2B‐YFP* revealed its expression restricted to flower meristem. Similarly, a significant number of cells showing *pAP1::H2B‐YFP* expression are present in the epidermis compared to the subepidermis and corpus cells (Figure 1bp). Taken together, 17 epidermal cell layer‐enriched TF genes were examined for their transcriptional activity. For eleven TF genes, we detected transcription activity in the epidermal cell layer in postembryonic development; however, six TF genes showed expression patterns beyond the epidermal cell layer.

### 2.5 Subepidermal cell layer TFs get activated postembryonically

**FIGURE 3:**
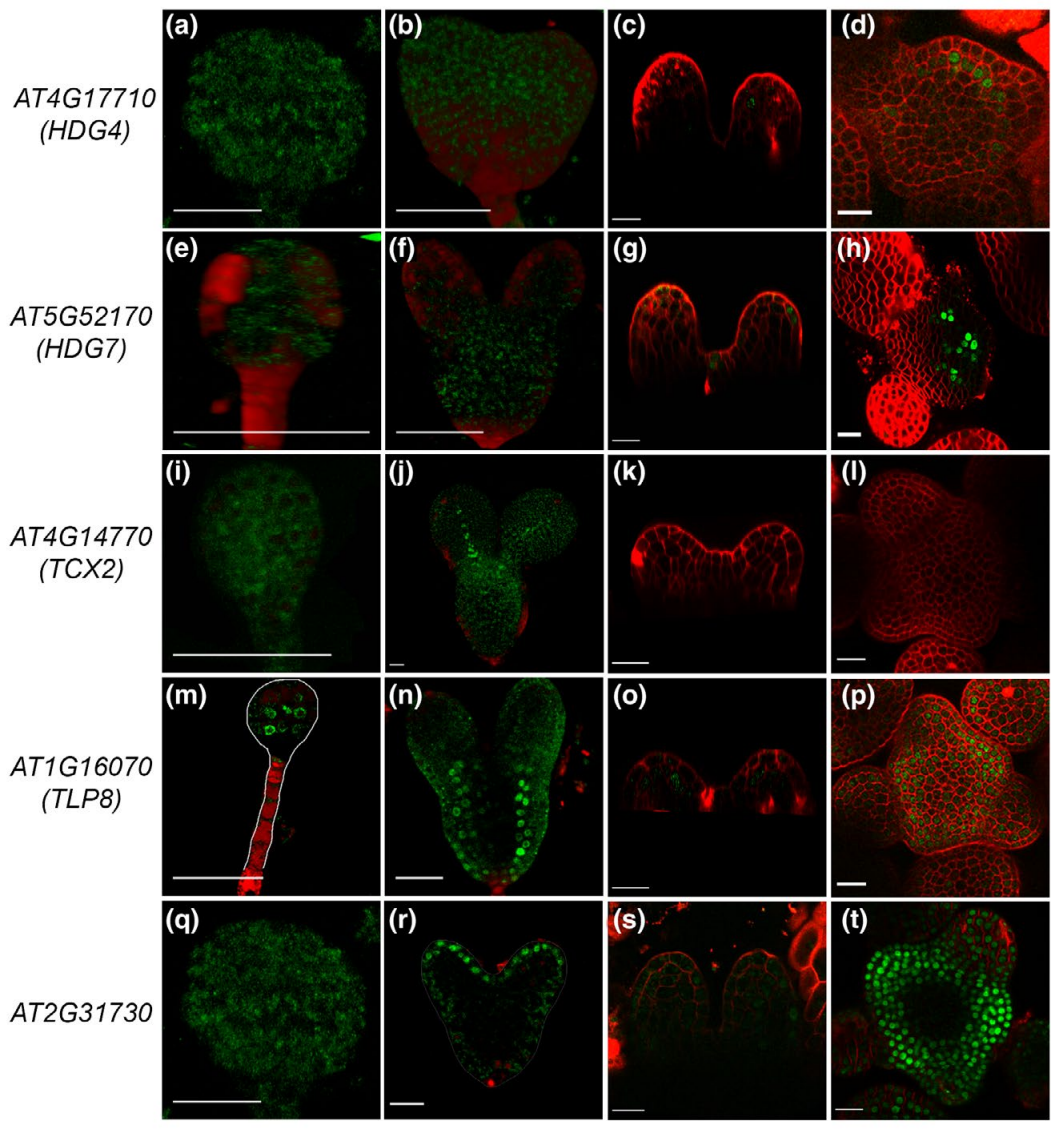
Subepidermal cell layer enriched TF genes display dynamic expression pattern. For each reporter line from left to right images represent embryonic and postembryonic stages of plant development. Mainly globular and heart stages of embryo development were investigated, whereas, for postembryonic development 3 DAG seedling and four‐week‐old IM were chosen (a–t). Interestingly, *HDG4* and *HDG7* show subepidermal cell layer specific expression in IM (d and h), in 3 DAG seedling expression of *HDG4* expression was sporadic and spotted in subepidermal cell (c), however, in *HDG7* it was variable and expressed both in epidermal and subepidermal cell layer (g). Scale bars = 20 μM

Epidermal cell identity is established in embryonic development. We argued that it would be logical to identify genes and their transcriptional activity restricted to the subepidermal cell layer in the SAM. Of the 21 subepidermal cell layer‐enriched TFs, promoter‐reporter constructs were analyzed for *HDG4*, *HOMEODOMAIN GLABROUS 7* (*HDG7*), *TUBBY LIKE PROTEIN 8* (*TLP8*), *TESMIN*/*TSO‐LIKE CXC2* (*TCX2*), *AT2G31730,* and *WRKY25* (Figure 3). Except for *TLP8*, none of the subepidermal cell layers‐enriched TF genes showed expression in the globular stage embryo (Figure 3a,e,i,m,q). *TCX2* and *LTP8* were found to be active in the presumptive vasculature cells in late torpedo and heart stage embryo, respectively (Figure 3j,n). Surprisingly, expression of *AT2G31730* was detected in the apical epidermis in the heart stage (Figure 3r).

HD‐ZIP family TFs, *HDG4,* and *HDG7* were found to be linked with subepidermis in 4‐week‐old SAM (Figure 3d,h); however, their expression was not visible in embryonic development (Figure 3a,b,e,f). In the 3‐day‐old seedlings, *HDG4* and *HDG7* expression start appearing in the epidermis and subepidermis cell sporadically, demonstrating that subepidermal cell identity is probably specified in plant postembryonic development (Figure 3c,d). *AT2G31730* and *TLP 8* are expressed broadly in the PZ cells but were missing from the CZ cells in SAM (Figure 3p,t). *TCX2* was found enriched in the subepidermis; however, the reporter did not show expression in the shoot apex, except in the epidermal cell layer of stage 5 sepal and leaf (Figure S2a–d). Taken together the data from six subepidermal cell‐enriched TF genes, except *HDG4* and *HDG7*, none of the genes supported the predicted expression patterns from Yadav et al., (2014), suggesting that there is a high probability of the subepidermal cell layer identity being specified in postembryonic development.

### 2.6 Validation of promoter‐reporter expression patterns

We compared the embryonic expression patterns of epidermal and subepidermal cell layers‐enriched TF transcriptional fusions with mRNA in situ hybridization and transcriptional fusions. For eight TF promoters, we confirmed expression in the early embryo (Abe et al., 2001, 2003; Nakamura et al., 2006) (Table S2). The reported expression patterns were also supported by the laser capture microdissection study by Casson et al., (2005). The gene expression studies do not support expression patterns of *pWRKY22* and *pAT1G75710*.

Of the 22 TF genes for which we successfully created transgenic lines and detected expression in IM. We confirmed the expression patterns for *HDG4*, *HDG5*, *ATML1*, *PDF2*, *HDG12*, *AP1*, *EGL3,* and *ATHMGB15* as reported in earlier studies (Abe et al., 2003; Alejandra Mandel et al., 1992; Nakamura et al., 2006; Yadav et al., 2009). The expression patterns detected for *HDG7*, *AtLTP8*, *ABF1*, *AT2G31730*, *CBF1*, *CAMTA6*, *AT5G54630*, *AT1G75710*, *NF‐YA5*, *PIL5*, *EIL1*, *WRKY11*, *WRKY17,* and *WRKY22* are supported in the IM by sorted cell type‐based gene expression study (Yadav et al., 2014). *pATH1* expression pattern did not match with the reported mRNA expression carried out by Gomez‐Mena and Sablowski (2008). To validate the expression pattern of *AT5G54630,* and *AT1G75710*, we carried out mRNA in situ hybridization on the tissue sections of 4‐week‐old shoot apices. The transcripts of both genes were restricted to the epidermal cell layer as reported in transcriptional fusions (Figure 1d, h; Figure S3a,b). *HDG7* expression was not detected by in situ hybridization perhaps due to low MAS5 expression scores (data not shown). Interestingly, *MYB94* and *ERF9* display high MAS5 score in epidermal cell population data, but their transcriptional fusions did not result in visible YFP expression in the epidermis. We conducted mRNA in situ hybridization experiment for *MYB94* and *ERF9*. In situ hybridization study revealed the enrichment of *MYB94* transcript in the epidermal cell layer, although a weak expression of *MYB94* was also noticed in the subepidermal cell layer (Figure S3d). *ERF9* expression was broad in the emerging flower primordia and IM (Figure S3c). We confirmed the expression of *CBF1* and *AT5G04760*/*DIVARICATA2* (Figure S3e, f). Taken together, we have determined expression patterns of four uncharacterized TF genes in shoot apex by in situ hybridization and validated expression patterns of *AT1G75710* and *AT5G54630*. In total, we have generated expression patterns for 26 epidermal and subepidermal cell layers‐enriched TF genes in the SAM.

### 2.7 Mapping of TF‐DNA interactions by enhanced yeast‐one‐hybrid (eY1H) assays

**FIGURE 4:**
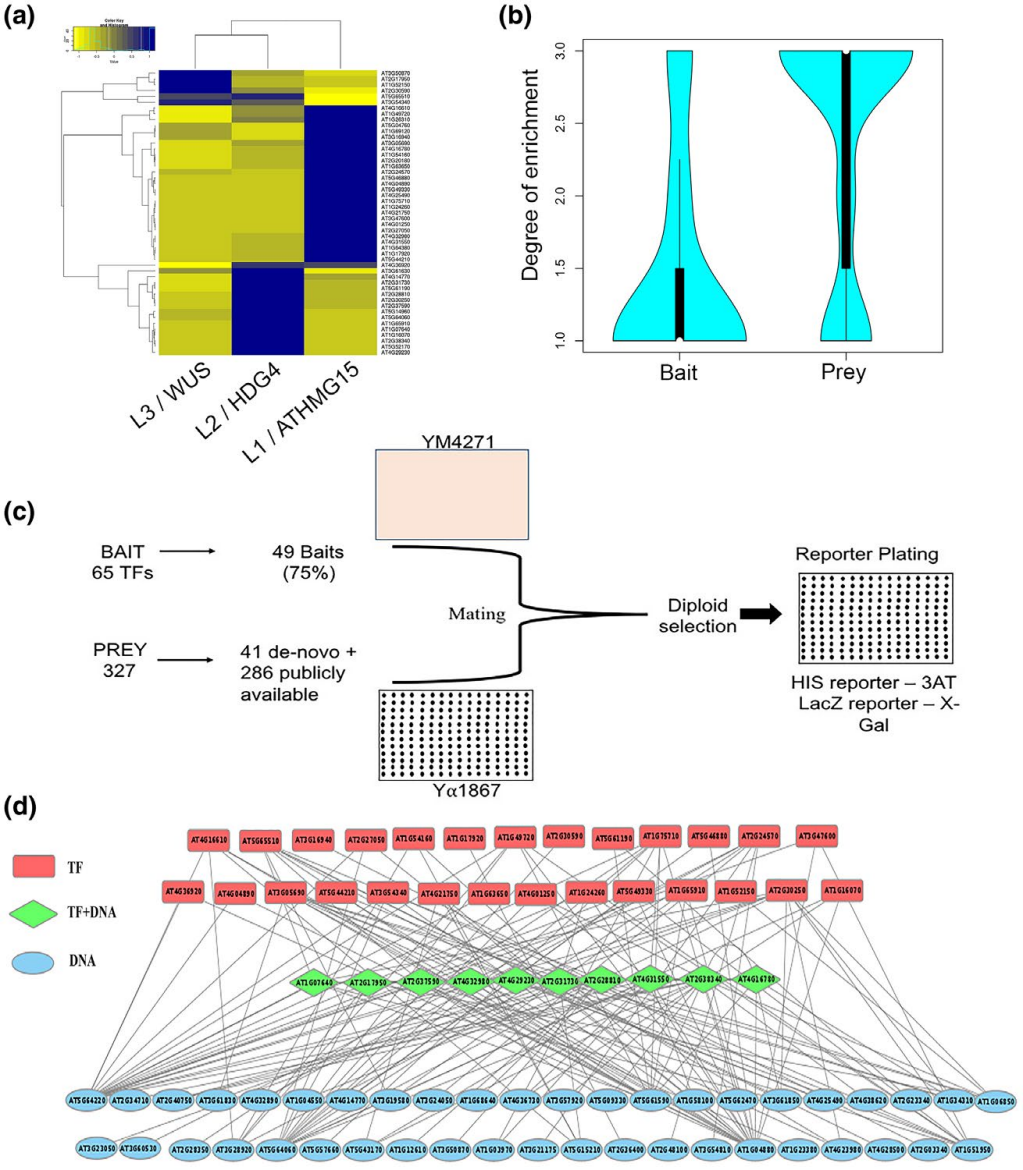
GRN of epidermal and subepidermal cell layer enriched TFs. Hierarchical clustering of 49 TF genes that are differentially expressed in SAM and used for making baits (a). Microarray data is derived from WUS, HDG4, and ATHMGB15 cell population of the shoot to plot the heatmap. Enrichment degree for bait and prey genes selected for eY1H was calculated (b). Experimental outline followed in conducting the eY1H assay is depicted (c), 49 TF promoters were used to make bait in YM4271 while 327 prey plasmids were transformed into Ya1867 (c). Bait and prey were mated, and diploids were selected on minimal media lacking histidine and tryptophan. A network of interactions deduced using eY1H is represented in the cytoscape (d)

In our effort to understand the dynamic regulation of epidermal and subepidermal cell layer‐enriched TF genes, we asked what controls the transcription of these genes in the first place. To delineate the regulatory network of epidermal and subepidermal TF genes, we took the 3‐kb promoter used for making reporter lines as bait and tested its interactions with the shoot derived TF prey proteins. From a library of ~700 TF prey clones (gifted by Siobhan Brady, UC Davis), we rescued 327 clones (Figure 4b and Table S3) and used for carrying out the eY1H screen. This library contains TFs that are expressed in various cell layers as well as in CZ, PZ, and RM cell types but having MAS5 normalization score (≥100) (Yadav et al., 2014). We used 3 kb upstream regulatory region of 41 TF genes to make the baits. The upstream 3 kb regulatory region of *APETALA2, APETALA3, ATHB‐15, MONOPOLE, PLETHORA7, SEPALLATA3, WRKY21,* and *WUSCHEL* were also included in this list (Figure 3a and Table S4). Taken together, 49 promoter fragments were assembled in the pMW2 vector as described in an earlier study to make the bait in yeast strain Ym4271 (Deplancke & Dupuy, 2004; Deplancke, Mukhopadhyay, et al., 2006; Gaudinier et al., 2011; Gubelmann et al., 2013; Taylor‐Teeples et al., 2015). TF prey plasmids were transformed into yeast strain Yα1867.

Using the Singer robot 16,023 interactions were setup (Gaudinier et al., 2011; Gubelmann et al., 2013), and we concluded GRN consisting of 165 interactions (edges) between 80 nodes (TFs and DNA elements) (Figure 4c,d, Figure S4, Table S5). Of the 80 nodes, 10 are both TFs and TF encoding gene promoters, 43 are TFs only, and 27 are TF encoding gene promoters. At least one interacting TF was found for 76% of the genomic regulatory elements. Moreover, more than one interacting partner was found for the majority of the promoters. A large number of baits had an in‐degree varying between 1 and 7. However, two baits, *DREB19* and *WRKY25* showed in‐degree of 10 or more (Figure S5a), suggesting that multiple upstream regulators can bind to a promoter or DNA element. Approximately 16% of the TFs are bound to at least one genomic regulatory element. For 49 TFs, the out‐degree varied between 1 and 7 (Figure S5b and Table S5). However, four TFs, DEWAX, ATHMGB15, CAMTA2, and ANAC103 exhibited out‐degree of more than10, suggesting that the TFs are involved in the regulation of multiple downstream targets and not solely dedicated for the regulation of a single‐target gene (Table S5). Hence, 53 TFs bind to 37 promoter baits in eY1H assay, comprising of 165 interactions. However, the number of interactions concluded in this network was relatively low because of the low coverage of baits and prey TF proteins. We have taken 27% (327 out of 1,225) of the shoot‐enriched TFs as prey. In *C. elegans*, 21,714 interactions were captured in a high‐throughput eY1H screen when 2,500 baits were tested against 366 TFs (Fuxman Bass et al., 2016), suggesting that by increasing the number of baits and preys, one can achieve many more functional relationships among the TFs and their target gene promoters. Future studies will explore all of the shoot derived TFs that are broadly expressed as preys to score all possible interaction with epidermal and subepidermal cell layer‐enriched TF gene promoters.

We next asked, what is the overlap between the native expression domains of the TFs and their target gene promoter. Microarray data from 10 shoot cell populations were used for this analysis (Yadav et al., 2009, 2014). The number of interactions falling at different bait and prey scores was plotted (Figure S6). In 22.4% of the interactions in the network, a complete overlap between the expression domain of the bait and the prey was observed. In 42.9% of the cases, the upstream regulator was found to be more broadly expressed, and in 25.6% of the cases, the target was found to be more broadly expressed than its interacting TF. Similar observations were made in the past for stele enrich TFs in the root (Brady et al., 2011).

### 2.8 Identifying the TF binding sites using FIMO

The likelihood of regulation will be very high of a target gene by an upstream TF if the promoter has at least one binding site in it. Of the 53 TFs, which are part of our GRN, we identified DNA motifs for 32 TFs using Finding Individual Motif Occurrences (FIMO) (Grant et al., 2011). Further, we were able to predict transcription factor binding sites (TFBS) for 29 TFs in at least one of the target gene promoter sequences using FIMO (*p* = 10^–4^). These twenty‐nine prey TFs participate in 101 interactions in the eY1H protein‐DNA interaction (PDI) network. Of the 101 interactions identified, 73 of them overlaps with FIMO prediction (Figure S7, and Table S6). For 28 interactions, FIMO was unable to assign the binding sites, suggesting that these interactions involved novel motifs, which are not discovered yet. Taken together, these analyses show that the gene‐centric approach of network building is not only efficient to capture the existing motif information but also can discover novel motifs not reported by earlier studies.

### 2.9 *In planta* validation of the protein‐DNA interaction network

**FIGURE 5:**
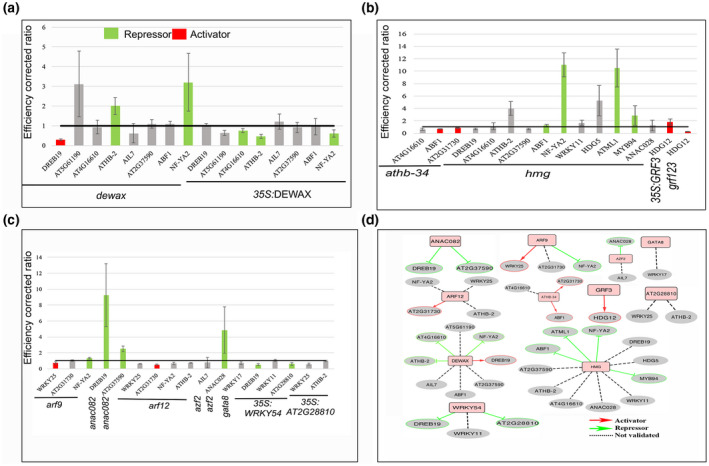
Validation of protein‐DNA interaction to construct subnetworks. Protein‐DNA interaction network tested for DEWAX in *dewax* mutant and overexpression lines (OX) (a). Colored bars indicate inferred relationship (e.g., red, activator; green, repressor and grey, not consistent). In (b) *athb34*, *athmgb15*, *grf123* mutant lines, and *35S::GRF3* OX used to test the target TFs. In (c) *arf9*, *arf12*, *azf2*, *gata8*, *annac082* mutant lines, and *WRKY54*, *AT2G28810* OX lines were used to infer the regulatory relationship with the targets. A summary of tested networks using RT‐qPCR assay (d). Red arrows indicate activating, and green lines indicate repressing relationships among the tested TFs. Dotted grey lines indicate non‐linear relationships

To determine whether the PDIs identified in yeast, occur *in planta,* we used reverse transcriptase quantitative PCR (RT‐qPCR) analysis to validate the GRN. To test this, T‐DNA insertion lines were used for isolating total RNA (Tables S7 and S8), and wherever overexpression lines were available, they were used for the validation of the network. For the DEWAX subnetwork, RT‐qPCR experiment was carried out in *dewax* mutant and *35S::DEWAX* overexpression lines. *DREB19* transcript levels were downregulated in the *dewax* mutant. However, in *35S::DEWAX*, the *DREB19* transcript levels did not alter significantly (Figure 5a). Apart from *DREB19*, DEWAX was also found to act as a repressor for *ATHB‐2*, *AT4G16610,* and *NF‐YA2* (Figure 5a). For the ARF9 subnetwork, three targets were identified *WRKY25, AT2G31730,* and *NF‐YA2*. ARF9 acts as an activator for *WRKY25,* while it represses *NF‐YA2* (Figure 5b). Another auxin response factor, ARF12 binds to the promoter of *WRKY25, AT2G31730, ATHB‐2,* and *NF‐YA2*. However, the subnetwork for ARF12 was validated only for *AT2G31730*. In *arf12* mutant, transcript levels of *AT2G31730* were reduced, indicating that ARF12 acts as an activator of *AT2G31730* (Figure 5b). ARF9 and ARF12 both bind to the promoter of *AT2G31730*. The likelihood of other ARF protein binding on the promoter of *AT2G31730* is not ruled out given the expression pattern of *pAT2G31730::H2B‐YFP* in the PZ of SAM in these lines. The *pAT2G31730* reporter is active in the periphery of SAM where usually the auxin signaling is high in comparison to CZ.

ANAC082 negatively regulates *DREB19* and *AT2G37590* (Figure 5b). Similarly, WRKY54 acts as a repressor for *DREB19* and *AT2G28810* (Figure 5b). Of the 11 interactors identified for the ATHMGB15 subnetwork in eY1H, four were negatively regulated, while the remaining seven could not be validated. *ABF1, MYB94, ATML1,* and *NF‐YA2* were regulated negatively by HMGB15 (Figure 5c). ATHB34 was found to be an activator for *ABF1* and *AT2G31730* (Figure 5c). AtGRF3 showed binding on *HDG12* promoter in eY1H assay and regulates it positively (Figure 5c).

We observed molecular phenotypes in 4 out of 8 (50%) DEWAX targets, 2 out of 3 (67%) ARF9 targets, 2 out of 3 (67%) for WRKY54 targets, 2 out of 2 (100%) for ANAC082 targets, 2 out of 3 (67%) for ATHB‐34 targets, 1 out of 2 (50%) for AZF2 targets, 1 out of 4 (25%) for ARF12 targets, 4 out of 11 (37%) for HMG targets, and 1 out of 1 (100%) for GRF3 target. None of the targets could be reproduced for GATA8 and AT2G28810. Of the 40 interactors tested, 19 were confirmed. Six of them get regulated positively by upstream factors, while 13 of them get regulated negatively by upstream regulators (Figure 5d). Remaining twenty‐one interactions did not show consistent results in the RT‐qPCR experiment. Taken together, 48% of targets showed molecular phenotype when tested *in planta,* confirming further the importance of the eY1H approach in delineating the Arabidopsis GRNs in the absence of high‐quality antibodies.

### 2.10 Inferring the function of putative TFs by T‐DNA analysis

To study the role of epidermal and subepidermal cell layer derived TFs, we analyzed the morphological phenotypes using loss and gain of function approaches. We identified T‐DNA insertion in 43 epidermal and sub‐epidermal‐enriched TF genes (Table S7). Of the 43T‐DNA lines analyzed, 16 lines showed complete loss of mRNA transcripts (Figure S8). To determine the morphological phenotypes of the epidermal and sub‐epidermal‐enriched TFs, we analyzed the T‐DNA insertion lines showing a significant reduction in the transcripts level of the given TF. We did not find any obvious phenotype that could indicate the function in the epidermal and subepidermal cell fate specification. Genetic redundancy in the function of epidermal cell layer‐enriched TF genes due to overlapping expression patterns perhaps has masked the developmental function.

## 3 DISCUSSION

### 3.1 Protoderm specific expression pattern is achieved by transcriptional repression

**FIGURE 6:**
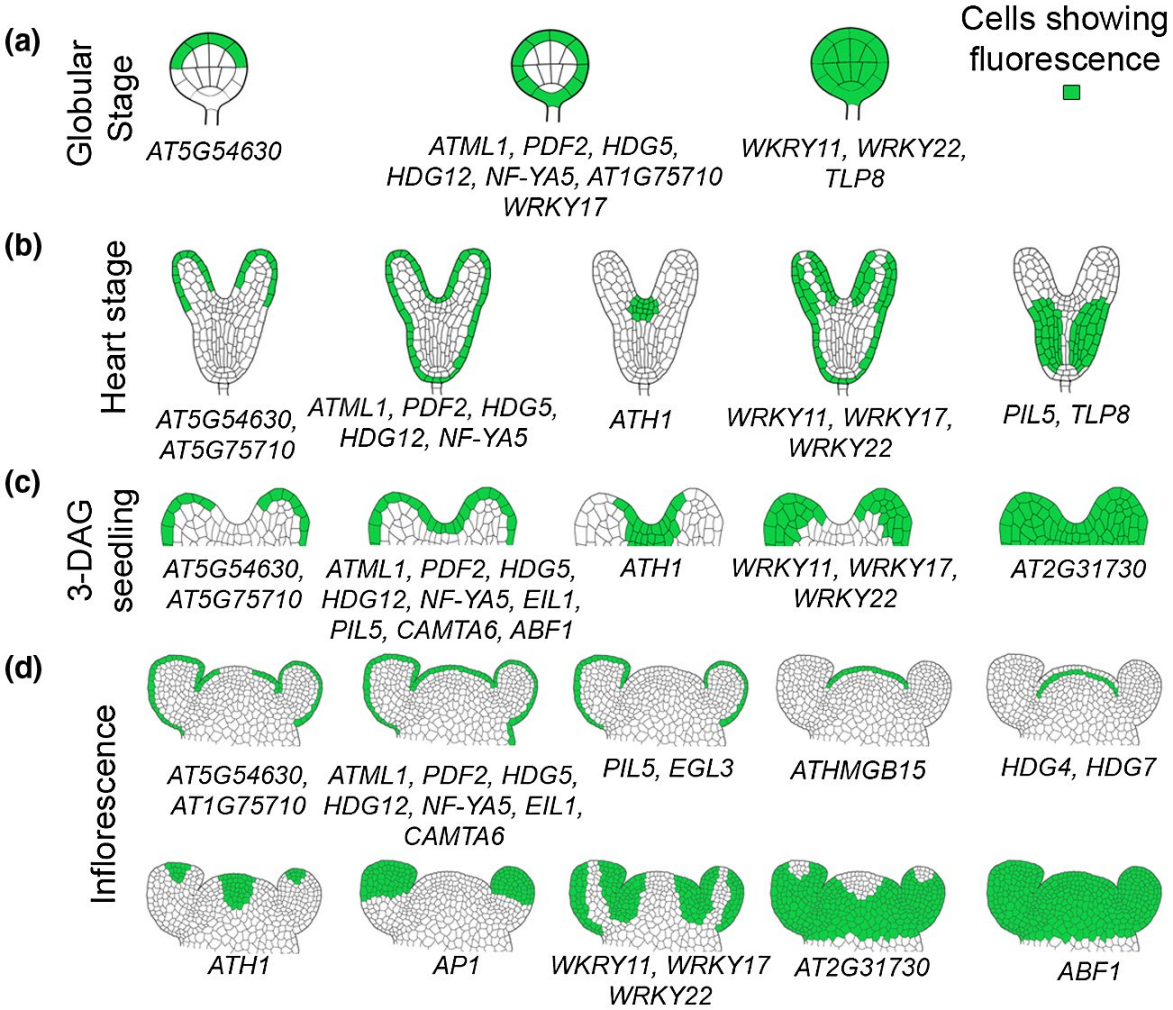
Schematic drawings of expression patterns captured for epidermal and subepidermal cell layers enriched TF genes. TFs expressed in globular stage (a), late heart stage (b), 3‐DAG seedlings (c), and 4‐week old IM (d). The Arabidopsis gene identifier and gene name are written below the representative expression pattern drawing

Here, we provide evidence how expression patterns arise for epidermal and subepidermal cell layer enriched TF genes in embryonic and postembryonic development of *Arabidopsis thaliana* (Figure 6 and Table S2). The extensive cataloguing followed with an analysis of expression patterns for seventeen epidermal cell layer‐enriched TF genes allowed us to classify them into three categories. For the first set of TF genes, promoter‐reporters clearly show expression in the epidermis as predicted by Yadav et al., (2014). We report expression of *AT5G75710*, *AT5G54630*, *EIL1*, *HDG5*, *HDG12*, *ATML1*, *PDF2*, *NF‐YA5*, *EGL3*, *CAMTA6,* and *PIL5* restricted to the epidermal cell layer. Protoderm specific expression pattern of *AT5G75710*, *HDG5*, *HDG12, PDF2,* and *NF‐YA5* closely resembles that of *ATML1* in the 16, 32, and 64 cell stage embryos (Figure 6). *NF‐YA5* expression is restricted to protoderm even before HD‐ZIP IV family TFs. The probability of specifying protoderm is very high in all cell layers similar to animal germ layers in early embryonic development in Arabidopsis. However, genes whose expression pattern is coupled with the protoderm identity are repressed in the inner cells of the globular stage embryo. The second set of TF genes were found to be enriched in the epidermal cell layer based on the cell population microarray study. But promoter‐reporter analysis shows that their expression is not confined to the epidermis. *ABF1*, *ERF9*, *WRKY11*, *WRKY17*, *WRKY22,* and *AP1* genes fall in this category. Interestingly, *ABF1* expression was observed everywhere in the young floral organ primordia but in the older buds, its expression gets restricted to the epidermis. For the third set of TF genes, promoter reporter constructs fail to show expression. The 3 kb promoter fragment taken here to generate reporter construct was not sufficient to drive the transcription because the critical cis‐elements required to achieve native expression were missing from the promoter. To achieve a native expression pattern for *CLV3* promoter reporter, besides 1.5 kb 5′ promoter element, a 1.2 kb 3′ promoter fragment was also added (Brand et al., 2002). A later study revealed the role of the 3′ promoter region in spatiotemporal regulation of *CLV3* gene in stem cells. This study showed that the activation of *CLV3* reporter gene in CZ cells is dependent upon the a cis‐regulatory module. This module is consists of a cluster of WUSCHEL binding sites, which are present within the 3′ promoter region of *CLV3* gene (Perales et al., 2016).

Of the 21 subepidermal cell layer TF genes, promoter‐reporter lines were generated for six. *pHDG4::H2B‐YFP* and *pHDG7::H2B‐YFP* showed an expression pattern linked to the subepidermis in reproductive SAM. *TLP8* and *AT2G31730* were confined PZ of the adult shoot, while *TCX2* did not show any expression. Surprisingly, *TCX2* expression was observed in the sepal and leaf epidermis (Figure S2). Our analysis of *HDG4* and *HDG7* promoter‐reporter lines revealed that the activity of H2B‐YFP translation fusion protein coincides with the subepidermis in adult SAM. In the early embryo, subepidermis linked expression patterns were not evident. In the 3‐DAG seedling, these promoters were found to be active both in epidermis and subepidermis. Later in the development, the pattern of expression becomes more clearer, and get confined to the subepidermis.

The promoter‐reporter data presented here for the epidermal and subepidermal cell layer‐enriched TF genes suggest the importance of stage and tissue‐specific upstream regulators in fine‐tuning the expression patterns in both embryonic and postembryonic development in Arabidopsis. Despite the complexity involved in the regulation of gene expression in multicellular plants, this study shows that in the 50% of reporter constructs cis‐elements present within the 3 kb 5′ upstream region is sufficient to recapitulate major patterns of transcription. For the remaining promoters, reporter construct did not show a spatiotemporal pattern because the cis‐elements required to achieve transcription are either present in introns or in the 3′ promoter region. Our work highlights the importance of using the regulatory elements beyond the 3 kb upstream promoter. In such cases, introns and 3′ promoter region could be combined in their order in the promoter‐reporter construct for the successful transcription.

### 3.2 A diverse set of upstream factors modulates the transcriptional activity of epidermal and subepidermal TF genes

To understand how cell‐type‐specific transcriptional outputs are initiated and maintained in Arabidopsis SAM. We used eY1H assay and generated PDIs for epidermal and subepidermal cell layers‐enriched TFs and their upstream regulators. A few baits showed a high level of auto‐activation in our assay and therefore could not be used for generating PDIs. Despite these limitations, eY1H provides a platform for high throughput studies and capture relevant regulators. eY1H assay may also produce false positives. That is why validation of the data is required using alternative approaches such as RT‐qPCR. Approximately 50% of the interactions tested in TF mutant or overexpression lines, caused changes in the expression of target genes. Similar results for PDIs were identified through eY1H screens in the past in *Drosophila melanogaster*, *C. elegans*, and *Arabidopsis thaliana* (Brady et al., 2011; Fuxman Bass et al., 2016; Hens et al., 2011; Sparks et al., 2016). Not all interactions captured in eY1H show molecular phenotypes in‐vivo because these may be false positives in eY1H or the interaction may be occurring in‐vivo but it might be neutral and may not have any regulatory effect (MacNeil et al., 2015).

Moreover, some regulations might have been missed or diluted because the whole seedling was taken for validation experiments. Whereas in vivo regulation might be occurring still at single‐cell level. Interactions discovered through eY1H assays may reflect functional significance if the binding sites for the interacting partner are enriched in the promoter of the target gene. However, the binding site enrichment alone may not reflect a regulatory relationship. Because binding site prediction methods rely only on the presence of predicted DNA motifs (White et al., 2013).

## 4 MATERIALS AND METHODS

### 4.1 Plant work and genetics

*Arabidopsis thaliana* Columbia‐0 (Col‐0) and Landsberg erecta (L*er)* ecotypes were used as WT strain. T‐DNA lines used in this study were obtained from ABRC. *grf123* triple mutant was a kind gift from Jeong Hoe Kim (Kyungpook National University, South Korea). Seeds were surface‐sterilized with 70% ethanol, followed by 4% (w/v) sodium hypochlorite (MERCK 1.93607.1021) containing 0.02% Triton X‐100 for 3‐min and rinsed three times with sterile distilled water. The seeds were sown on Murashige and Skoog (MS) medium containing 0.8% Bacto agar (HiMedia, India), 1% (w/v) sucrose, and 0.1% (w/v) MES. Stratified seeds were kept in darkness at 4°C for three days and then transferred to plant growth chambers (Conviron, Canada and Percival Scientific, USA). For transformation, WT L*er* dry seeds were sown on soilrite mix (KELTECH Energies Ltd.) and treated at 4°C for three days and then placed in the growth chambers (Conviron PGC Flex) under Philips fluorescent tube lights (120 µmol light and 22°C) on a long day cycle (16 hr light and 8 hr dark). The soil was prepared by mixing soilrite mix (KELTECH Energies Ltd.), compost, and perlite in the ratio of 3:1:1/2 as described in Saini et al. (2020).

## CONFLICT OF INTEREST

The authors declare no competing financial interests.

## AUTHOR CONTRIBUTIONS

R.Y. conceived the project, S.B., H.K., R.Y. designed all experiments. S.B., H.K., M.M., P.S., S.Y., and S.Y. performed the experiments. S.B., H.K., S.K.S., J.K., and R.Y. analyzed the data. S.B., H.K., J.K., and R.Y. wrote the paper.

## Supporting information

Fig S1‐S8

Table S1‐S11

Methods
