# Supplementary material for: A cellular expression map of epidermal and subepidermal cell layer‐enriched transcription factor genes integrated with the regulatory network in Arabidopsis shoot apical meristem
Source: Plant Direct. 2021 Mar 18;5(3):e00306. doi: 10.1002/pld3.306 (PMC7970154; doi:10.1002/pld3.306)
Supplement: Supplementary file 1 — Fig S1‐S8 [file PLD3-5-e00306-s002.docx]

**Supplemental Figures**

**
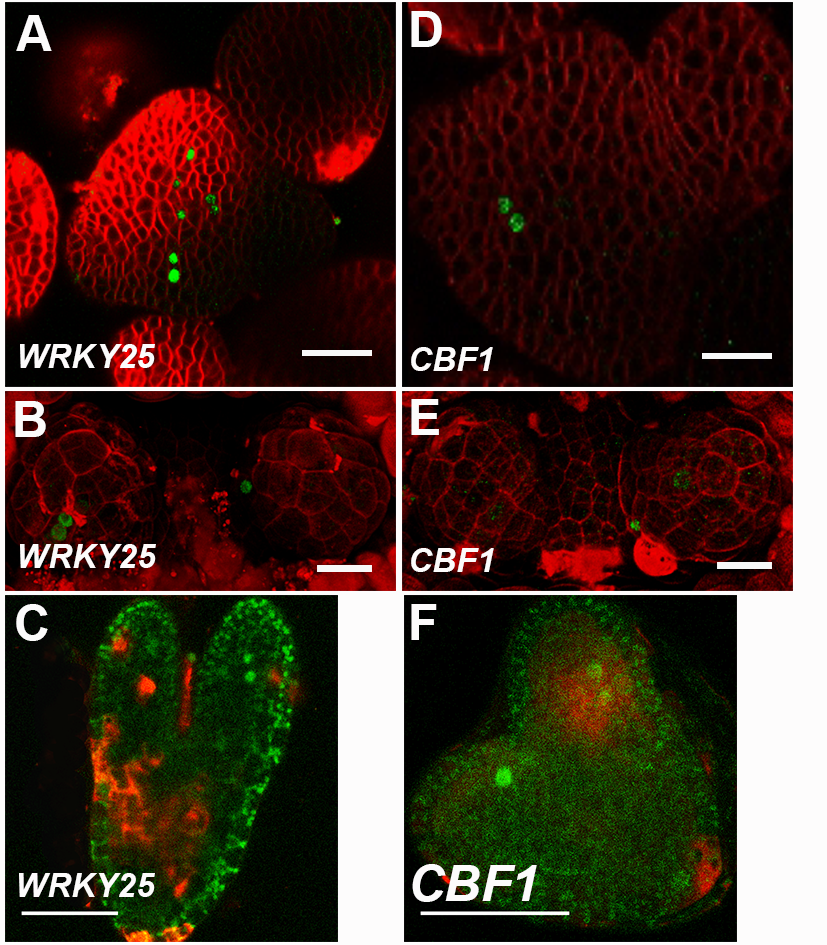
**

**Figure S1.** Inconsistent expression of *pWRKY25* and *pCBF*. *pWRKY25* is active in SAM, 3 DAG seedling and torpedo stage embryo (A-C). *pCBF1* expression in SAM, 3 DAG seedling and heart stage embryo (D-F). Both the promoter reporter construct showed sporadic expression in the SAM, 3 DAG seedling and in embryo. Scale bars = 20µM.

**
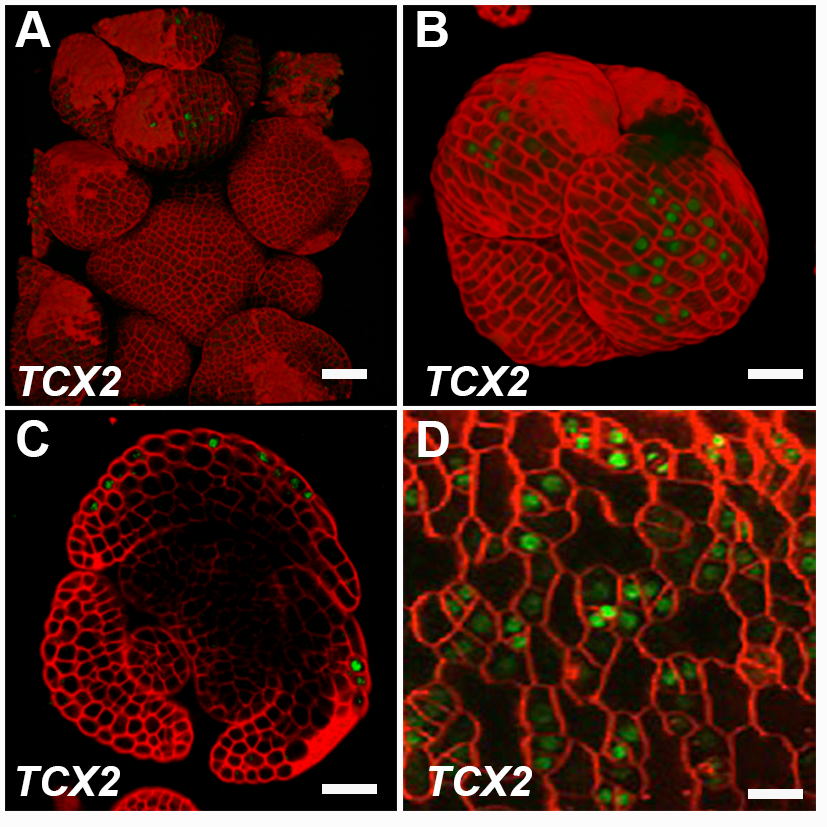
**

**Figure S2.** *TCX2* expression in inflorescence and in pavement cells. The reconstructed top view of SAM using optical slices revealing the L1 layer in (A) and stage 5 flower in (B). Optical section of (B) in (C), and *TCX2* expression in the leaf epidermis (D). Pavement cells do not show expression of *TCX2*. Scale bars = 20 µM.


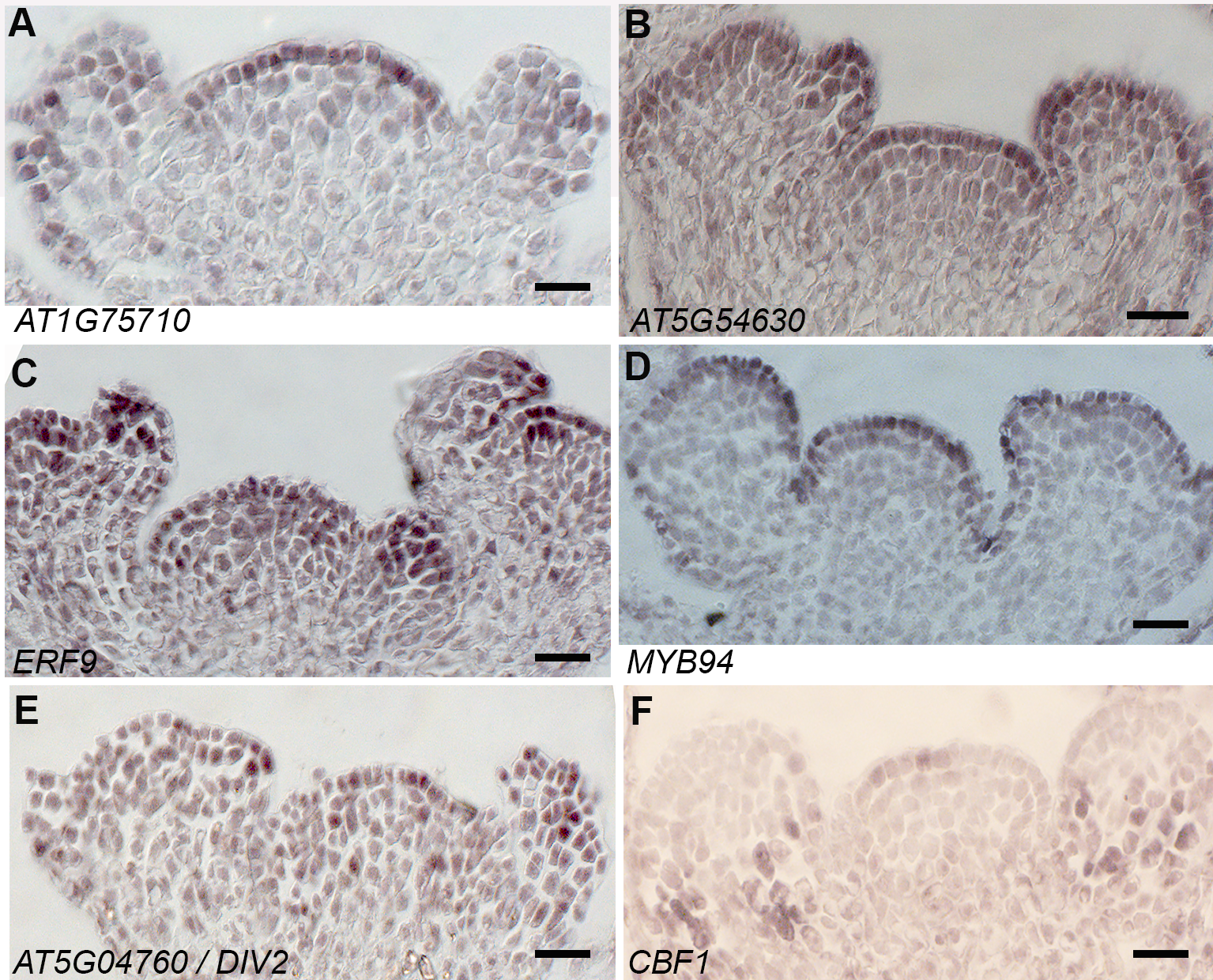


**Figure S3.** **Expression patterns of *AT1G75710*, *AT5G54630*, *ERF9*, *MYB94*, *DIV2* and *CBF1* observed in inflorescence meristem.** The expression pattern observed for AT1G75710 and AT5G54630 are similar to reporter construct (A and B). ERF9 is expressed in the young flower and lateral organs (C). *MYB94* is expressed in the epidermal cell layer followed with weak expression in subepidermal cell layer (D). *DIV2* and *CBF1* both showed patch expression (E and F). *pCBF1* showed a similar expression as shown in (Fig. S1D). Scale bars = 20 µM.


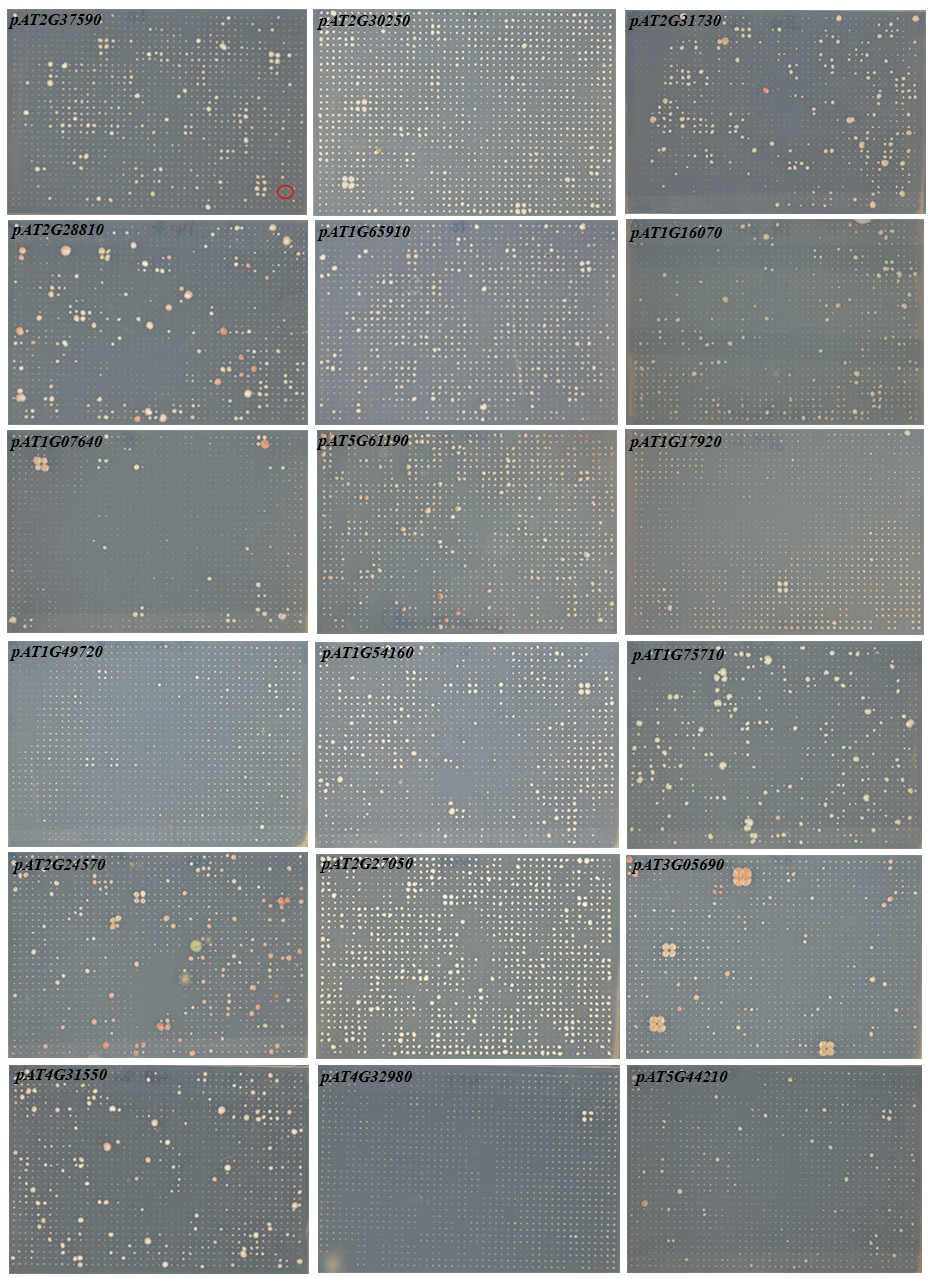


**Figure S4. Yeast-1-hybrid robotic screen using *HIS* auxotrophic marker.** Representative images of plates spotted using SINGER ROBOT for various bait constructs after mating in the Yeast-one-hybrid screen.


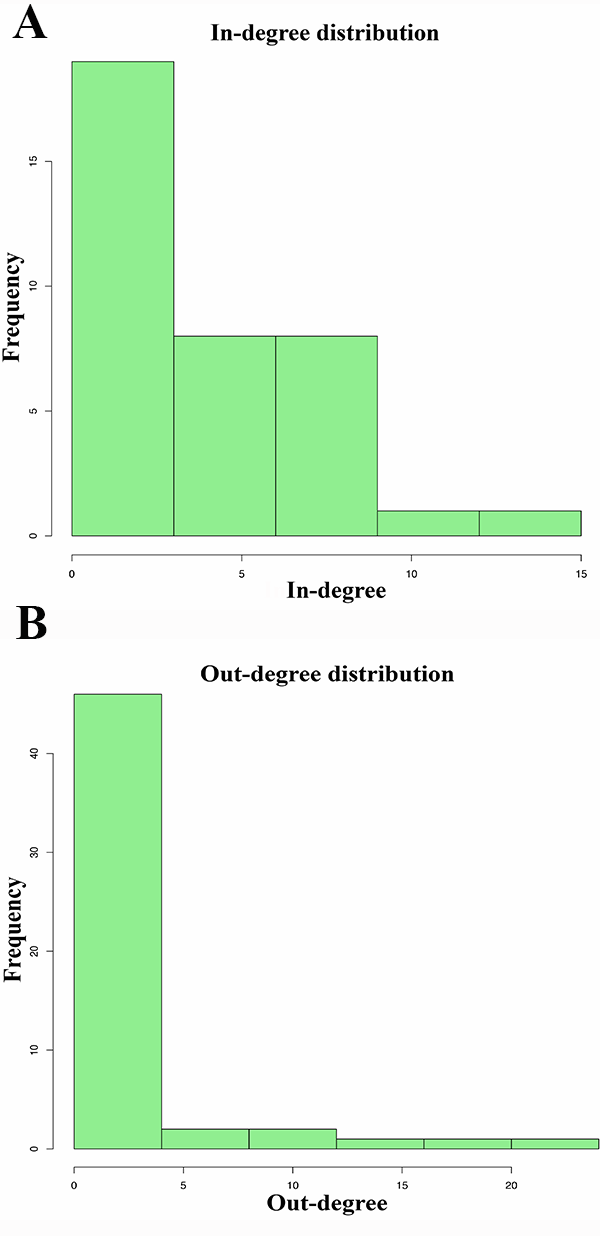


**Figure S5. In-degree and Out-degree distribution.** (A), (B) Histogram graphs are representing In-degree and Out-degree distribution property of the network, respectively. X-axis represents the degree (number of connections) and Y-axis represents the number of nodes associated with that degree.


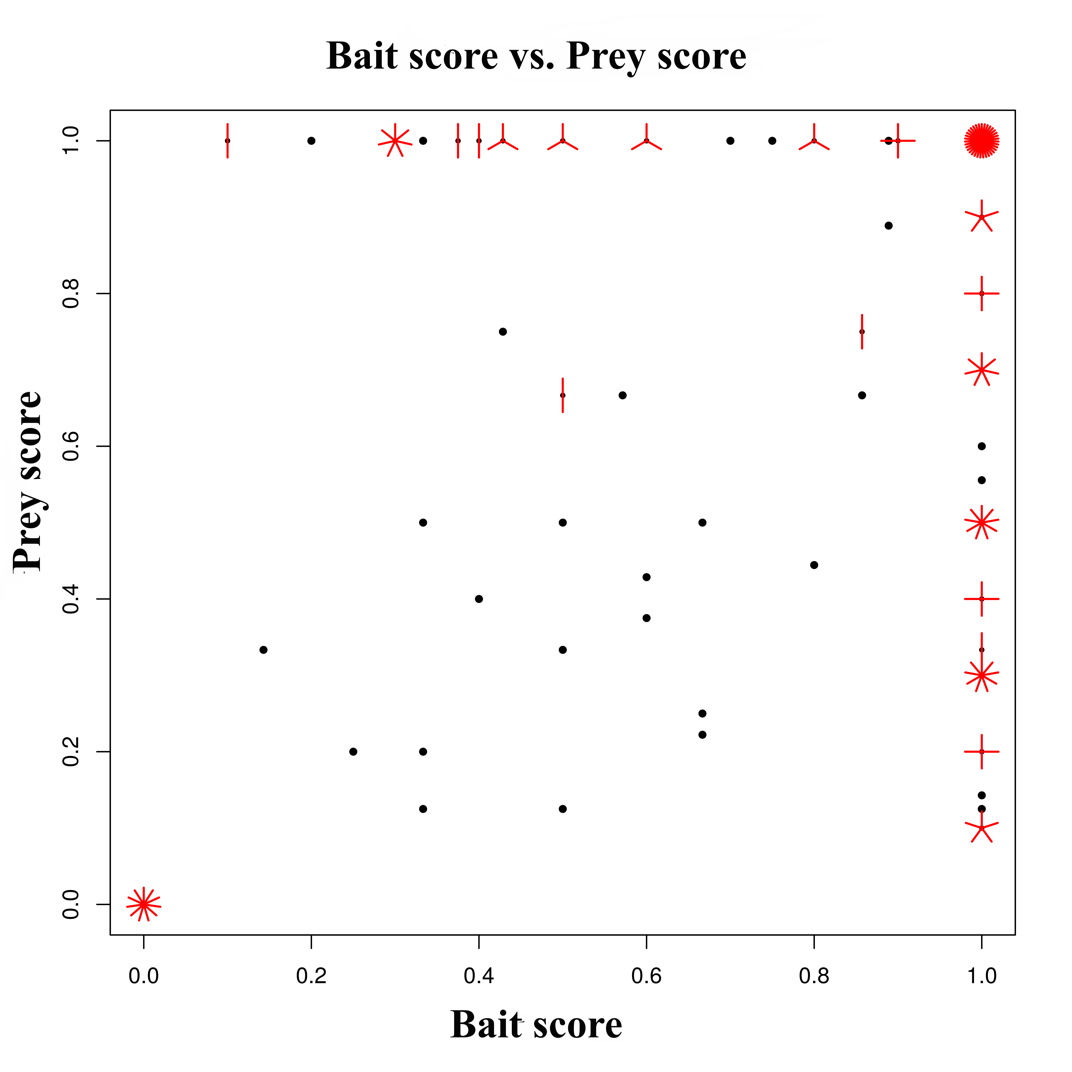


**Figure S6. Expression overlap between bait and prey expression.** Sunflower plot representing the number of interactions falling at different bait and prey scores. For 22.4% of interactions, bait and prey show identical expression domain. In 42.9% of the cases, the upstream regulator was broadly expressed. In 25.6% of the cases, the target was found to be more broadly expressed.


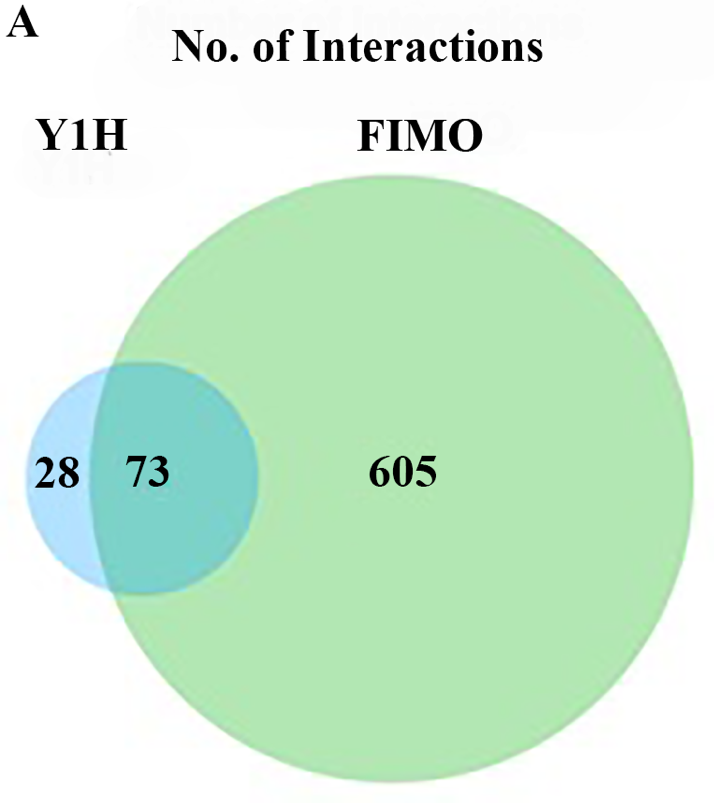


**Figure S7. The overlap between eY1H interactions and predicted interactions.** Venn diagram showing the overlap between the number of interactions predicted vs. observed at p-value <= 1E-4. 1073 interactions were possible between 29 TFs and 37 DNA elements. At stringent cut-off of 1E-4, ~72.2% of the eY1H interactions have a binding prediction in FIMO.


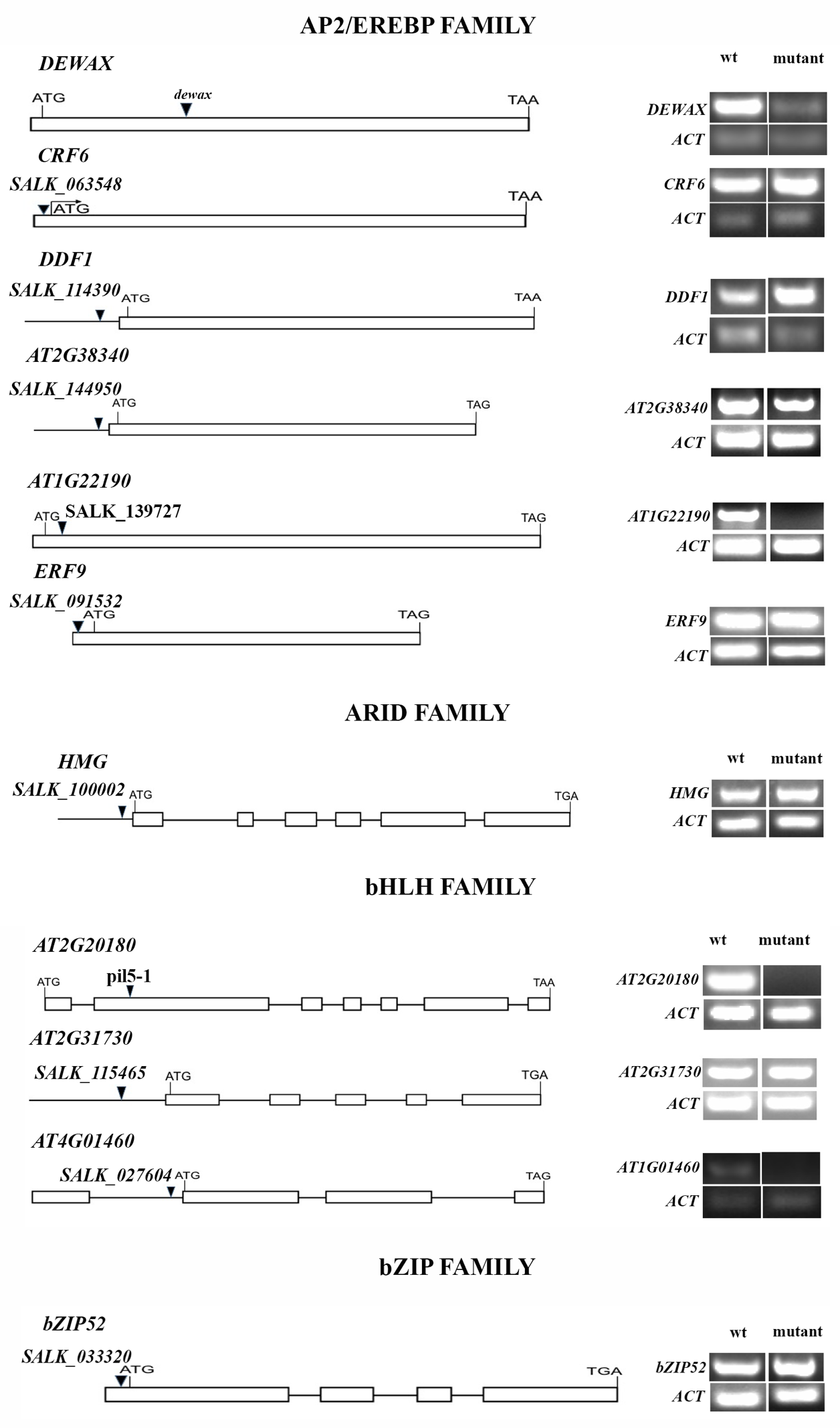


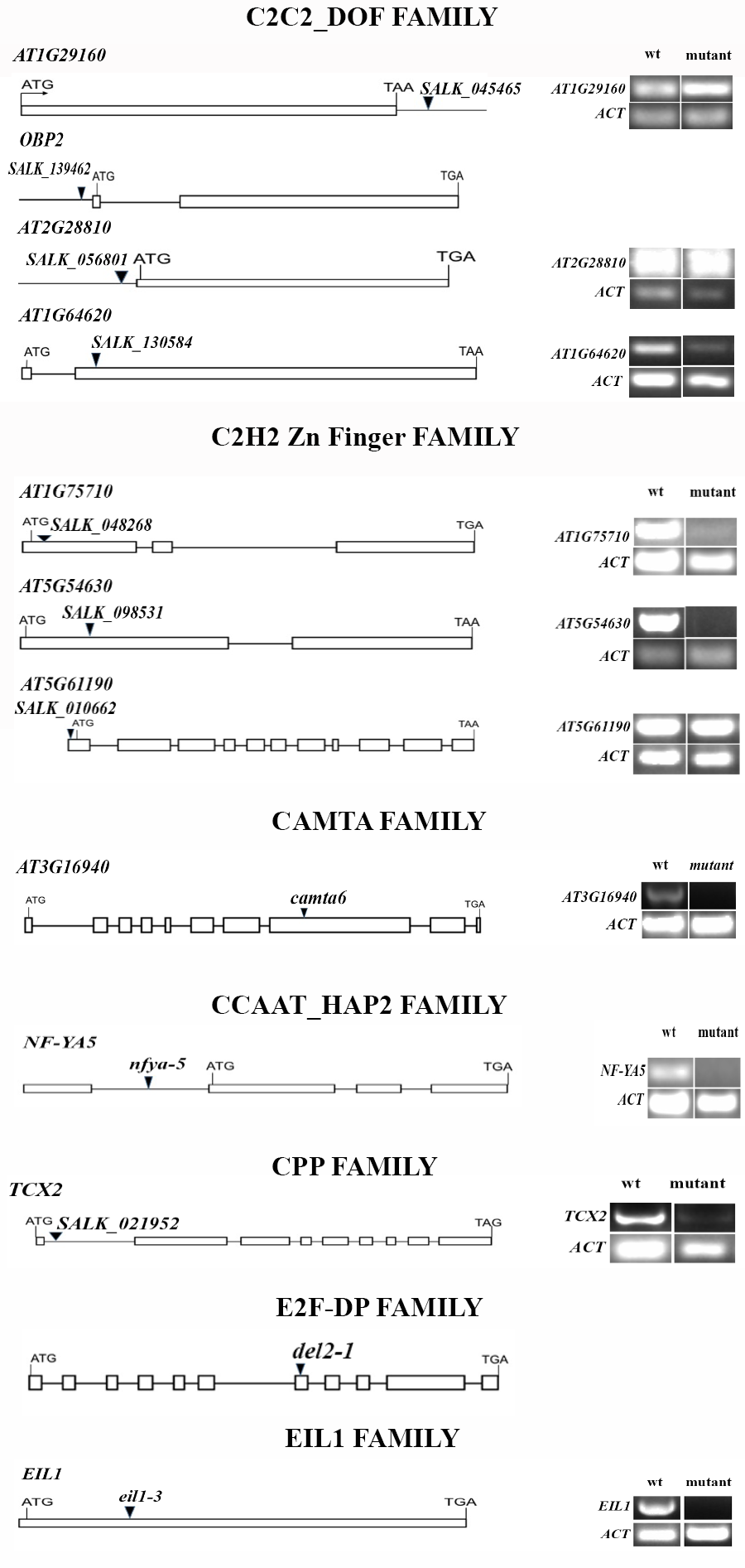


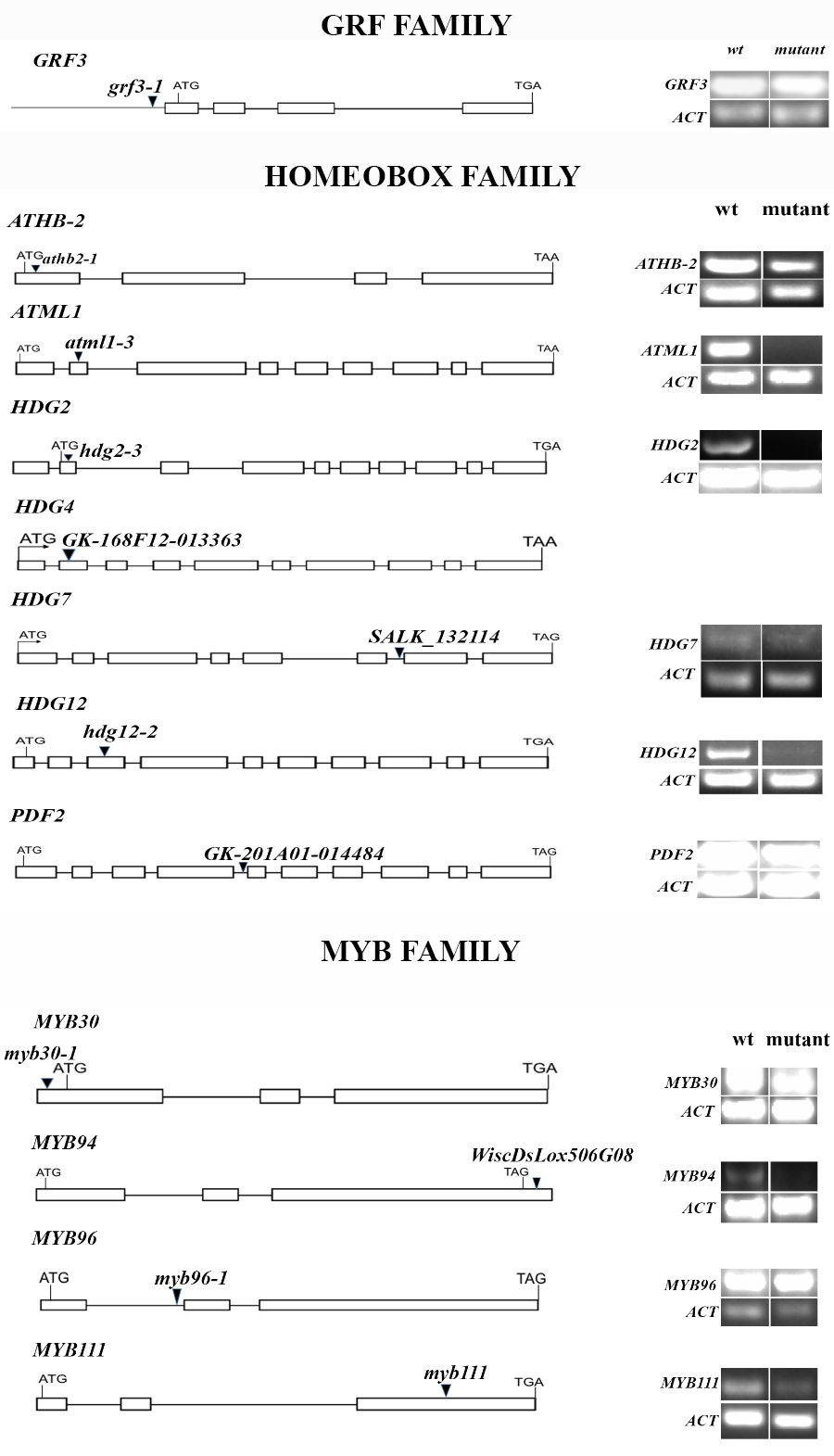


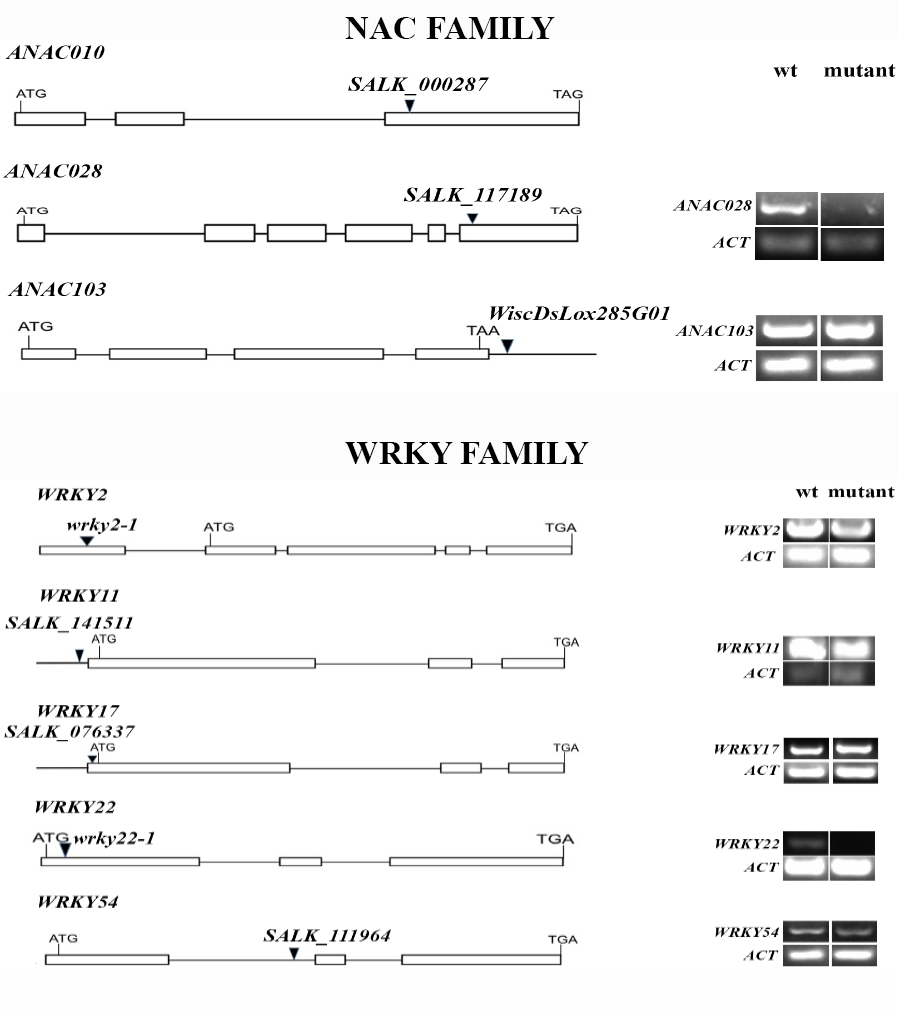


**Figure S8. Characterization of T-DNA insertion lines for epidermal and subepidermal cell layer enrich TFs.** Genomic structures of *Arabidopsis* TF family genes, enriched in the SAM and locations of T-DNA insertions. Boxes indicate exons, whereas lines indicate introns. Arrowheads indicate the position of T-DNA insertion in the given gene. Alongside are shown the RNA levels detected in mutant vs wild type in semi-quantitative reverse transcript PCR. *ACTIN* was used as an internal control.
