# Supplementary material for: A cellular expression map of epidermal and subepidermal cell layer‐enriched transcription factor genes integrated with the regulatory network in Arabidopsis shoot apical meristem
Source: Plant Direct. 2021 Mar 18;5(3):e00306. doi: 10.1002/pld3.306 (PMC7970154; doi:10.1002/pld3.306)
Supplement: Supplementary file 3 — Methods [file PLD3-5-e00306-s003.docx]

**Online supplementary information**

**Materials and Methods**

**Promoter reporter constructs and transgenic lines**

For generating promoter::H2B-YFP transcriptional fusion constructs, promoters of 29 TF genes were PCR amplified using the respective bait pDONR vector as a template and cloned into pENTR/D/TOPO (Supplemental Table S1 and S4). Later on, LR clonase reaction was set up between the respective pENTR clone and pGreen 0229 destination vector as reported in Yadav et al., (2014). To perform the LR recombination reaction directly using pDONRP4P1-R bait vector into plant transformation vector, we cloned the attR4:ccdB:attR1 cassette from pMW2 vector into compatible sites of pGreen 0229. This modified vector was used for generating 16 promoter::H2B-YFP transcriptional fusion constructs. All binary promoter::H2B-YFP fusion vectors were introduced into *Agrobacterium* GV3101 and were transformed into WT L*er* background by floral dip transformation (Clough and Bent, 1998). Transformed plants (T1) were selected on soil using BASTA (BAYER Crop Sciences, Germany) and screened for expression using an upright epifluorescence microscope (Supplemental Table S1).

**TF open reading frame cloning for making preys**

Total RNA was isolated from shoot apices of WT L*er* plants using QIAGEN RNeasy kit. First strand cDNA synthesis was carried out by Superscript III First Strand cDNA synthesis kit (Invitrogen, USA). Forward primers containing a stretch of four nucleotides (CACC) towards the 5’ end was designed to amplify the full-length CDS and cloned into pENTR/D/TOPO vector. The cloned CDS of selected TFs were sequenced fully to confirm the integrity of the insert. A number of cDNA clones were obtained either from the Arabidopsis Biological Resource Centre (ABRC, Ohio, USA) or from RIKEN (RIKEN, Japan). All TF ORFs without stop codon were PCR amplified using a forward primer having 5’ CACC overhang and reverse primer, respectively, and cloned into pENTR/D/TOPO vector (sequence information is listed in Supplemental Table S3 and S10). The resulting pENTR clones were recombined with pDEST-AD-2µ using LR clonase II, and translation fusion between AD and CDS was sequence verified.

**TF DNA bait cloning**

To amplify the TF promoter fragments, WT L*er* genomic DNA was used as a template. For epidermal and subepidermal cell type enriched TFs, 3kb upstream regulatory region from the translational start site of the gene was amplified using forward and reverse primers, respectively (Supplemental Table S4). The amplicons were recombined with the pDONRP4P1-R vector using BP clonase II. Resulting pENTRY clones were recombined with pMW2 (*HIS3* reporter vector) and pMW3 destination vectors (*LacZ* reporter vector) (Deplancke et al., 2006) using LR clonase reaction, respectively. For genomic integration of DNA bait-reporter constructs, 3µl linearized vector DNA was added into 15µl aliquot of competent yeast cells of YM4271 (a kind gift from Siobhan Brady, University of California Davis, USA). Yeast transformation was performed using 40% polyethylene glycol (PEG). Transformants were selected on selective media plates lacking either histidine (for *HIS3* reporter) or both histidine & uracil (for *HIS3* and *LacZ* reporter). *HIS3* reporter showed high reproducibility in our robotic assay in comparison to *LacZ* reporter. Furthermore, we noticed that the LacZ reporter did not yield any color reaction where *HIS3* based reporter clearly showed growth. Therefore, most of the interactions in this study are concluded based on the HIS3 reporter, as reported in an earlier study (Gubelmann et al., 2013).

**Prediction of binding sites using Finding Individual Motif Occurrences** **FIMO**

To decode the functional relationship between the upstream regulators and the target gene promoters, we collated TF binding sites data, which were identified previously using ChIP-ChIP, ChIP-seq, DAP-seq, amp-DAP, SELEX and protein binding microarray (PBM). High quality DNA motifs were extracted in the form of Position Weight Matrix (PWM) for individual TFs from the following databases; i) PlantCistromeDB (O’Malley et al., 2016), ii) CIS-BP (Weirauch et al., 2014), iii) JASPAR (Khan et al., 2018) and iv) PBM (Franco-Zorrilla et al., 2014). After collating the information from all the databases, we ended up with 1870 redundant motifs, which corresponds to at least 682 unique TFs. We also used 612 motifs from the Plant Transcription Factor Database for this purpose (Jin et al., 2017).

3 kb promoter sequences used as DNA baits were scanned for the presence of TF binding sites using the FIMO package (Grant et al., 2011) with a p-value cut-off of 10^-4^. The upstream 3kb sequences of the TFs used as baits were used for calculating the background nucleotide frequency. Only TF promoters that were involved in an interaction in the eY1H assay were used for scanning in FIMO (Supplemental Table S6). To find out whether the motifs identified in our promoters using FIMO are by chance or have significance over random enrichment. PDI network was randomized 25000 times by preserving the topology of the network; i.e. keeping the in-degree and out-degree of the nodes same and the overlap in the interactions between eY1H PDI network. FIMO predictions were counted every time. The distribution had a mean of 65.40876000 and standard deviation of 3.57596354. Therefore, the number of overlapping interactions observed between eY1H assay and FIMO predictions are significant (*P* =0.016885).

**Plasmid construct and generation of transgenic lines**

To make *35S::GRF3* overexpression construct, *GRF3* CDS was PCR amplified and cloned into pENTR/D/TOPO. A gateway LR-reaction was set up with pMDC32. It was introduced in the WT to generate the overexpression lines. Similarly, miR396 resistant version of *GRF3* was engineered by introducing a synonymous mutation in miR396 binding motif of *GRF3* region in pENRT/D/TOPO as described in the Debernardi et al., (2014). The resulting *rGRF3* version was used to set up LR-clonase reaction with pMDC32 to make *35S::rGRF3*. The CDS of *AT2G28810*, *WKRY54*, and *DEWAX* were PCR amplified and cloned into pENTR/D/TOPO vector. After verifying the sequence, an LR reaction was set up with pMDC32 destination vector with respective entry clone. The resulting vectors carrying CDS of interest were transformed by the floral dip method, and transgenic plant lines were raised and made homozygote for the respective transgene. The primer sequence is given in Supplemental Table S9.

**Mating based eY1H assays**

pMW2 and pMW3 constructs carrying the promoter of interest were integrated into the yeast genome, and transformants were selected on media lacking histidine and uracil, respectively. Promoter reporter integration into the yeast genome was first verified by PCR and then followed with sequencing. AD-TF encoding plasmids were transformed into the Yα1867 strain (a kind gift from Siobhan Brady, University of California Davis, USA) and transformants were selected on media lacking tryptophan, and further were allowed to grow for two-days at 30^o^C. Each prey colony was spotted four times to create a 1536 array format of transformed yeast cells on Sc-Trp plates with RoToR HAD robot (Singer Instrument, UK). Glycerol stock of prey strain was used to set up new biological replicate every time. Bait strains were spread as a lawn on separate YPD plates. For mating, first AD-TF colonies were spotted on a YPD plate and on top of them a given DNA bait strain was printed. Mating plates were left overnight at 30^o^C, and then the mated diploids were transferred on fresh plates lacking both histidine and tryptophan with RoToR HAD robot. These plates were allowed to grow for two days at 30^o^C. To score the interactions, diploid yeast colonies were transferred onto Sc-His -Trp plates having varying 3-AT concentrations (5-40mM). These plates were usually analyzed after 5-7 days of incubation at 30^o^C. Positive interactions were concluded by activation of *HIS3* (by addition of 3-aminotriazole in the media) and *LacZ* (by addition of X-Gal in the media) reporters. Every interaction was set up in quadruplicate on the plate. eY1H assay was carried out by setting up biological replicates three times. Interactions that showed growth at least twice in three replicates were considered as positive.

**Laser scanning confocal microscopy**

For each promoter reporter construct, inflorescence meristem of T1 plants carrying promoter::H2B-YFP transgene was dissected after bolting under a dissecting microscope (Leica, Germany). Plants expressing the nuclear localized H2B-YFP were identified under an upright epifluorescence microscope using long working distance water dipping 63X objective (Zeiss, Germany). We identified several independent insertions for each promoter reporter and used expression pattern from two representative lines for comparison with microarray data (Supplemental Table S1). YFP was excited at 515 nm wavelength with argon laser lines at 10-15% laser power. The YFP emission spectra were filtered using 530-550 nm variable bandpass filter. To mark the cell outline, FM4-64 (10µg / ml, Thermofisher) or Propidium iodide (PI, 10 µg/ml, Sigma) was applied directly to the dissected shoot apex 10-15 minutes before imaging. Confocal image stacks of 30-40 sections spaced ~ 1.5 µm apart with scan speed of 400 milliseconds, 512 X 512-pixel / frame were collected using long working distance water dipping 63X objective (SP8 upright confocal microscope Leica, Germany). Both the YFP and FM4-64 were excited using the 515 nm laser line in conjunction with adjustable bandwidth filters of 530-550 nm and 600-650 nm.  Whenever PI was used for staining the cell outline, it was excited using a 561 nm laser line, and emission spectra was filtered with 600-650 nm adjustable bandpass filter. Rest of the settings were kept as described above.

The lines established based on the inflorescence meristem imaging were used for embryo and seedling imaging. Siliques of appropriate age with immature seeds were dissected on a glass slide in distilled water. Ovules were stripped from the ovary wall and placed into FM4-64FX (Invitrogen) on a microscope slide. Embryos were popped out with the help of insulin syringes under a dissecting scope, and a coverslip was placed over the isolated embryos and sealed with nail polish. Imaging was carried out immediately under a 63X oil immersion objective in Leica SP8 upright confocal microscope as described above.

For seedling shoot apex imaging, seeds were surface sterilized with bleach and kept in the dark at 4^o^C for 3 days after putting on MS media plates. Plates were then kept vertically in the growth chamber for 3 days at 22^o^C. Germinated seedlings at their earliest stages (mostly with unopened/semi opened cotyledons) were chosen for imaging and transferred on Magenta boxes containing 1.5% solidified agar. Seedlings were pushed into the pre-created holes into agar leaving their top outside. Shoot apical meristem was exposed by gently removing one cotyledon with the help of fine tweezers (5TI, Dumont, Switzerland) and oriented vertically to visualize under the upright confocal nose piece. Propidium iodide (10µg/ml, Invitrogen) drops were put on the top of dissected seedlings and kept for 30-40 sec to visualize the cell outline. Autoclaved distilled water was poured over the seedlings to submerge, and z-stacks were taken by Leica SP8 confocal microscope equipped with a long-distance water dipping lens (63X objective). Rest of the confocal microscope settings were followed as described above.

**In situ hybridization**

TF coding sequences cloned into pENTR/D/TOPO vector (Invitrogen) were PCR amplified as a template for probe synthesis. All other steps were followed as per the protocol posted (<http://www.its.caltech.edu/~plantlab/html/protocols.html)> as previously described (Yadav et al., 2009; Yadav et al., 2010). CDS cloned into pENTR/D/TOPO vector for making eY1H prey plasmid for respective genes were used as templates. To synthesize the full-length sense probe, first, the template was PCR amplified with the help of primer pair, SP/pENTR-D-TOPO/ T7, and ASP/pENTR-D-TOPO/T7. The PCR amplified probe template carrying a T7 promoter sequence was used for making sense probe after purification for in vitro transcription reaction. Similarly, antisense probe template strand was synthesized with the help of primer pair, SP/pENTR-D-TOPO /antisense and ASP/pENTR-D-TOPO/antisense, and thus the resulting PCR product carries a T7 promoter sequence that can be used for setting in vitro transcription reaction to synthesize the antisense probe. The primer name along with sequence is given in Table S9.

**T-DNA mutant analysis**

T-DNA insertion lines for epidermal and subepidermal enriched TFs, and TFs that showed interactions with our bait of interest in the GRN were obtained from Arabidopsis Biological Resource Center (ABRC, Ohio, USA). For SALK, SAIL and WISCONSIN lines, primers were designed using iSect Primer design tool (<http://signal.salk.edu/tdnaprimers.2.html)>. For GABI-KAT insertion lines, the primer sequence information was either obtained directly from the website (<https://www.gabi-kat.de/db/primerdesign.php)> or designed using the GK primer tool.  Genomic DNA was isolated using a modified CTAB method (Murray and Thompson, 1980). Segregating insertion lines were confirmed for homozygosity by comparing the results of T-DNA-gene amplification with gene specific amplification using Columbia-0 as wild type control.  Plants having T-DNA insertion in both the chromosome did not show any gene specific amplification (Figure S8).  Hence, we determined T-DNA insertion in the homozygous state in 45 TF encoding genes. The complete list of T-DNA insertion mutant analyzed and primer sequence information related to T-DNA genotyping in this study is provided along with AGI in Supplemental Table S7.

**RT-qPCR validation of Y1H interactions**

Total RNA was isolated from 5-day old wild type and homozygous mutant line seedlings, using the ReliaPrep RNA tissue miniprep kit (Promega, USA). First strand cDNA was synthesized from 2µg total RNA as input using iScript cDNA synthesis kit (BioRad, USA). Gene expression was measured for two biological replicates using at least three technical replicates for mutant and wild type pair. Standard curves were run for each primer pair and values represented by the efficiency corrected quantification model as described by Sparks et al., (2016) (Supplemental Table S8 and S11). RT-qPCR experiments were carried out using a BioRad CFX6 machine (BioRad, USA). PP2A was used as an internal control in these experiments. Primer sequences information is described in Table S11.

To validate GRF3-HDG12 regulatory node, epidermal cells were sorted from *35S::GRF3* plant line crossed with *pHDG12::HDG12-EGFP* line and total RNA was isolated from ~100,000 epidermal cells. For control, cells were sorted from plants harboring *pHDG12::HDG12-EGFP* constructs. RT-qPCR experiment revealed the up regulation of *HDG12* transcripts in the epidermal cell layer in response to *GRF3* overexpression.

**Percentage overlap between preys and baits**

To calculate how much overlap bait has with an interacting prey or vice versa in terms of their expression. A gene was counted to be expressed in a particular cell population dataset if the gene gets a “P” (present call). For each biological replicate, PMA detection call was made using the MAS5 algorithm (Gautier et al., 2004). For every interacting pair, the percentage expression overlaps of bait with the prey (bait score) and prey with the bait (prey score) were calculated based on the PMA calls. Bait score is defined as the number of cell types in which both bait and prey are expressed divided by the number of cell types in which only the bait is expressed. Similarly, prey score is defined as the number of cell types in which both prey and bait are expressed divided by the number of cell types in which only prey is expressed. The number of interactions having different bait and prey scores was then plotted.

**References**

**Clough SJ, Bent AF** (1998) Floral dip: A simplified method for Agrobacterium-mediated transformation of *Arabidopsis thaliana*. Plant J **16**: 735–743

**Debernardi JM, Mecchia MA, Vercruyssen L, Kaufmann K, Inze D, Rodriguez RE** (2014) Post ‐ transcriptional regulation of GRF transcription factors by microRNA miR396 and GIF co ‐ activators controls leaf size and longevity. Plant J.

**Deplancke B, Mukhopadhyay A, Ao W, Elewa AM, Grove C a, Martinez NJ, Sequerra R, Doucette-Stamm L, Reece-Hoyes JS, Hope I a, et al** (2006) A gene-centered C. elegans protein-DNA interaction network. Cell **125**: 1193–205

**Franco-Zorrilla JM, López-Vidriero I, Carrasco JL, Godoy M, Vera P, Solano R** (2014) DNA-binding specificities of plant transcription factors and their potential to define target genes. Proc Natl Acad Sci. doi: 10.1073/pnas.1316278111

**Gautier L, Cope L, Bolstad BM, Irizarry RA** (2004) Affy - Analysis of Affymetrix GeneChip data at the probe level. Bioinformatics. doi: 10.1093/bioinformatics/btg405

**Grant CE, Bailey TL, Noble WS** (2011) FIMO: Scanning for occurrences of a given motif. Bioinformatics. doi: 10.1093/bioinformatics/btr064

**Gubelmann C, Waszak SM, Isakova A, Holcombe W, Hens K, Iagovitina A, Feuz JD, Raghav SK, Simicevic J, Deplancke B** (2013) A yeast one-hybrid and microfluidics-based pipeline to map mammalian gene regulatory networks. Mol Syst Biol. doi: 10.1038/msb.2013.38

**Jin J, Tian F, Yang DC, Meng YQ, Kong L, Luo J, Gao G** (2017) PlantTFDB 4.0: Toward a central hub for transcription factors and regulatory interactions in plants. Nucleic Acids Res. doi: 10.1093/nar/gkw982

**Khan A, Fornes O, Stigliani A, Gheorghe M, Castro-Mondragon JA, Van Der Lee R, Bessy A, Chèneby J, Kulkarni SR, Tan G, et al** (2018) JASPAR 2018: Update of the open-access database of transcription factor binding profiles and its web framework. Nucleic Acids Res. doi: 10.1093/nar/gkx1126

**Murray MG, Thompson WF** (1980) Rapid isolation of high molecular weight plant DNA. Nucleic Acids Res **8**: 4321–4326

**O’Malley RC, Huang S-SC, Song L, Lewsey MG, Bartlett A, Nery JR, Galli M, Gallavotti A, Ecker JR** (2016) Cistrome and Epicistrome Features Shape the Regulatory DNA Landscape. Cell. doi: 10.1016/j.cell.2016.04.038

**Sparks EE, Drapek C, Gaudinier A, Li S, Ansariola M, Shen N, Hennacy JH, Zhang J, Turco G, Petricka JJ, et al** (2016) Establishment of Expression in the SHORTROOT-SCARECROW Transcriptional Cascade through Opposing Activities of Both Activators and Repressors. Dev Cell. doi: 10.1016/j.devcel.2016.09.031

**Weirauch MT, Yang A, Albu M, Cote AG, Montenegro-Montero A, Drewe P, Najafabadi HS, Lambert SA, Mann I, Cook K, et al** (2014) Determination and inference of eukaryotic transcription factor sequence specificity. Cell. doi: 10.1016/j.cell.2014.08.009

**Yadav RK, Girke T, Pasala S, Xie M, Reddy GV** (2009) Gene expression map of the Arabidopsis shoot apical meristem stem cell niche. Proc Natl Acad Sci U S A **106**: 4941–6

**Yadav RK, Tavakkoli M, Reddy GV** (2010) WUSCHEL mediates stem cell homeostasis by regulating stem cell number and patterns of cell division and differentiation of stem cell progenitors. Development **137**: 3581–9

**Yadav RK, Tavakkoli M, Xie M, Girke T, Venugopala RG** (2014) A high-resolution gene expression map of the arabidopsis shoot meristem stem cell niche. Dev. doi: 10.1242/dev.106104
